# Novel multiple sclerosis susceptibility loci implicated in epigenetic regulation

**DOI:** 10.1126/sciadv.1501678

**Published:** 2016-06-17

**Authors:** Till F. M. Andlauer, Dorothea Buck, Gisela Antony, Antonios Bayas, Lukas Bechmann, Achim Berthele, Andrew Chan, Christiane Gasperi, Ralf Gold, Christiane Graetz, Jürgen Haas, Michael Hecker, Carmen Infante-Duarte, Matthias Knop, Tania Kümpfel, Volker Limmroth, Ralf A. Linker, Verena Loleit, Felix Luessi, Sven G. Meuth, Mark Mühlau, Sandra Nischwitz, Friedemann Paul, Michael Pütz, Tobias Ruck, Anke Salmen, Martin Stangel, Jan-Patrick Stellmann, Klarissa H. Stürner, Björn Tackenberg, Florian Then Bergh, Hayrettin Tumani, Clemens Warnke, Frank Weber, Heinz Wiendl, Brigitte Wildemann, Uwe K. Zettl, Ulf Ziemann, Frauke Zipp, Janine Arloth, Peter Weber, Milena Radivojkov-Blagojevic, Markus O. Scheinhardt, Theresa Dankowski, Thomas Bettecken, Peter Lichtner, Darina Czamara, Tania Carrillo-Roa, Elisabeth B. Binder, Klaus Berger, Lars Bertram, Andre Franke, Christian Gieger, Stefan Herms, Georg Homuth, Marcus Ising, Karl-Heinz Jöckel, Tim Kacprowski, Stefan Kloiber, Matthias Laudes, Wolfgang Lieb, Christina M. Lill, Susanne Lucae, Thomas Meitinger, Susanne Moebus, Martina Müller-Nurasyid, Markus M. Nöthen, Astrid Petersmann, Rajesh Rawal, Ulf Schminke, Konstantin Strauch, Henry Völzke, Melanie Waldenberger, Jürgen Wellmann, Eleonora Porcu, Antonella Mulas, Maristella Pitzalis, Carlo Sidore, Ilenia Zara, Francesco Cucca, Magdalena Zoledziewska, Andreas Ziegler, Bernhard Hemmer, Bertram Müller-Myhsok

**Affiliations:** 1 Max Planck Institute of Psychiatry, 80804 Munich, Germany.; 2 Munich Cluster for Systems Neurology (SyNergy), 81377 Munich, Germany.; 3 Department of Neurology, Klinikum rechts der Isar, Technical University of Munich, 81675 Munich, Germany.; 4 Central Information Office KKNMS, Philipps University Marburg, 35043 Marburg, Germany.; 5 Department of Neurology, Klinikum Augsburg, 86156 Augsburg, Germany.; 6 Department of Neurology, University of Leipzig, 04103 Leipzig, Germany.; 7 Institute of Medical Microbiology, Otto-von-Guericke University, 39120 Magdeburg, Germany.; 8 Department of Neurology, St. Josef Hospital, Ruhr-University Bochum, 44791 Bochum, Germany.; 9 Department of Neurology, Focus Program Translational Neurosciences (FTN) and Research Center for Immunotherapy (FZI), Rhine-Main Neuroscience Network (rmn^2^), University Medical Center of the Johannes Gutenberg University Mainz, 55131 Mainz, Germany.; 10 Department of Neurology, University Hospital Heidelberg, 69120 Heidelberg, Germany.; 11 Department of Neurology, University of Rostock, 18147 Rostock, Germany.; 12 NeuroCure Clinical Research Center, Department of Neurology, and Experimental and Clinical Research Center, Max Delbrück Center for Molecular Medicine, and Charité University Medicine Berlin, 10117 Berlin, Germany.; 13 Institute of Clinical Neuroimmunology, Ludwigs-Maximilians-Universität, 81377 Munich, Germany.; 14 Department of Neurology, Hospital Köln-Merheim, 51109 Köln, Germany.; 15 Department of Neurology, University Hospital Erlangen, 91054 Erlangen, Germany.; 16 Department of Neurology, Klinik für Allgemeine Neurologie, University of Münster, 48149 Münster, Germany.; 17 Clinical Neuroimmunology Group, Department of Neurology, Philipps-University of Marburg, 35043 Marburg, Germany.; 18 Department of Neurology, Hannover Medical School, 30625 Hannover, Germany.; 19 Institute of Neuroimmunology and Multiple Sclerosis and Department of Neurology, University Medical Centre Hamburg-Eppendorf, 20251 Hamburg, Germany.; 20 Department of Neurology and Translational Center for Regenerative Medicine, University of Leipzig, 04103 Leipzig, Germany.; 21 Department of Neurology, University of Ulm, 89081 Ulm, Germany.; 22 Neurological Clinic Dietenbronn, 88477 Schwendi, Germany.; 23 Department of Neurology, Medical Faculty, Heinrich Heine University, 40225 Düsseldorf, Germany.; 24 Neurological Clinic, Medical Park, 65520 Bad Camberg, Germany.; 25 Department of Neurology and Stroke, and Hertie Institute for Clinical Brain Research, Eberhard-Karls-Universität Tübingen, 72076 Tübingen, Germany.; 26 Institute of Computational Biology, Helmholtz Zentrum München, 85764 Neuherberg, Germany.; 27 Institute of Human Genetics, Helmholtz Zentrum München, 85764 Neuherberg, Germany.; 28 Institut für Medizinische Biometrie und Statistik, Universität zu Lübeck, Universitätsklinikum Schleswig-Holstein, Campus Lübeck, 23562 Lübeck, Germany.; 29 Institute of Human Genetics, Technische Universität München, 81675 Munich, Germany.; 30 Department of Psychiatry and Behavioral Sciences, Emory University, Atlanta, GA 30329, USA.; 31 Institut für Epidemiologie und Sozialmedizin der Universität Münster, 48149 Münster, Germany.; 32 Lübeck Interdisciplinary Platform for Genome Analytics, Institutes of Neurogenetics and Integrative and Experimental Genomics, University of Lübeck, 23562 Lübeck, Germany.; 33 School of Public Health, Faculty of Medicine, Imperial College London, SW7 2AZ London, UK.; 34 Institute of Clinical Molecular Biology, Kiel University, 24105 Kiel, Germany.; 35 Research Unit of Molecular Epidemiology, Helmholtz Zentrum München, 85764 Neuherberg, Germany.; 36 Institute of Epidemiology II, Helmholtz Zentrum München, 85764 Neuherberg, Germany.; 37 Institute of Human Genetics, University of Bonn, 53127 Bonn, Germany.; 38 Department of Biomedicine, Division of Medical Genetics, University of Basel, 4031 Basel, Switzerland.; 39 Interfaculty Institute for Genetics and Functional Genomics, Ernst Moritz Arndt University and University Medicine Greifswald, 17475 Greifswald, Germany.; 40 Institute of Medical Informatics, Biometry, and Epidemiology, University Hospital Essen, University Duisburg-Essen, 45122 Essen, Germany.; 41 Department I of Internal Medicine, Kiel University, 24105 Kiel, Germany.; 42 Institute of Epidemiology and Biobank popgen, Kiel University, 24105 Kiel, Germany.; 43 Institute of Genetic Epidemiology, Helmholtz Zentrum München, 85764 Neuherberg, Germany.; 44 Department of Medicine I, Ludwig-Maximilians-Universität, 81377 Munich, Germany.; 45 DZHK (German Centre for Cardiovascular Research), partner site Munich Heart Alliance, 80802 Munich, Germany.; 46 Institute of Clinical Chemistry and Laboratory Medicine, University Medicine Greifswald, 17475 Greifswald, Germany.; 47 Department of Neurology, University Medicine Greifswald, 17475 Greifswald, Germany.; 48 Institute of Medical Informatics, Biometry, and Epidemiology, Chair of Genetic Epidemiology, Ludwig-Maximilians-Universität, 81377 Munich, Germany.; 49 Institute for Community Medicine, University Medicine Greifswald, 17475 Greifswald, Germany.; 50 Istituto di Ricerca Genetica e Biomedica, Consiglio Nazionale delle Ricerche, Monserrato, 09042 Cagliari, Italy.; 51 Dipartimento di Scienze Biomediche, Università degli Studi di Sassari, 07100 Sassari, Italy.; 52 Center for Advanced Studies, Research and Development in Sardinia (CRS4), Pula, 09010 Cagliari, Italy.; 53 Zentrum für Klinische Studien, Universität zu Lübeck, 23562 Lübeck, Germany.; 54 School of Mathematics, Statistics, and Computer Science, University of KwaZulu-Natal, Pietermaritzburg, Scottsville 3209, South Africa.; 55 Institute of Translational Medicine, University of Liverpool, Liverpool L69 3BX, UK.; 56 Department of Neurology, University Hospital Bern and University of Bern, 3010 Bern, Switzerland.

**Keywords:** Multiple sclerosis, genome-wide association study, DNA methylation, L3MBTL3, MAZ, ERG, DLEU1, SHMT1

## Abstract

We conducted a genome-wide association study (GWAS) on multiple sclerosis (MS) susceptibility in German cohorts with 4888 cases and 10,395 controls. In addition to associations within the major histocompatibility complex (MHC) region, 15 non-MHC loci reached genome-wide significance. Four of these loci are novel MS susceptibility loci. They map to the genes *L3MBTL3*, *MAZ*, *ERG*, and *SHMT1*. The lead variant at *SHMT1* was replicated in an independent Sardinian cohort. Products of the genes *L3MBTL3*, *MAZ*, and *ERG* play important roles in immune cell regulation. *SHMT1* encodes a serine hydroxymethyltransferase catalyzing the transfer of a carbon unit to the folate cycle. This reaction is required for regulation of methylation homeostasis, which is important for establishment and maintenance of epigenetic signatures. Our GWAS approach in a defined population with limited genetic substructure detected associations not found in larger, more heterogeneous cohorts, thus providing new clues regarding MS pathogenesis.

## Teaser

Genome-wide study in Germans identifies four novel multiple sclerosis risk genes and confirms already known gene loci.

## INTRODUCTION

Multiple sclerosis (MS) is an autoimmune disease of the central nervous system. Human leukocyte antigen (*HLA*) alleles, located within the major histocompatibility complex (MHC) region, have been identified as major genetic determinants for the disease (*1*, *2*). In addition, more than 100 non-MHC MS susceptibility variants have been described (*3*, *4*). Many of the genes carrying known susceptibility variants are involved in the regulation of either immune cell differentiation or signaling (*4*–*8*). However, because the heritability of MS is limited (*9*), environmental contributions to disease etiology are also important (*10*). Environmental influences can alter gene expression via epigenetic mechanisms (*11*). Epigenetic alterations, such as DNA methylation or histone modifications, have been observed in tissues and cells of MS patients (*8*, *12*–*14*). Nevertheless, the impact of epigenetic regulation in MS is not yet understood.

The known genetic variants outside the MHC region have predominantly been established in large international collaborative studies. To achieve large sample sizes with the power to detect associations, these studies have combined sample sets from diverse ethnic populations (*4*–*6*). So far, the variants affecting MS susceptibility identified in these studies account for only 25% of disease heritability under an additive model of heritability (*3*), warranting for additional studies to fully unravel the genetic contribution to disease susceptibility. In contrast to the previously investigated large international cohorts, we have strived to examine the genetic contribution to MS susceptibility in a more homogeneous population, focusing entirely on German cases and controls. The genetic substructure among Germans is low (*15*). We therefore expected to have sufficient power to detect novel associations with moderate effect sizes in a data set showing little population stratification. Using a total of 4888 cases and 10,395 controls, we had 80% power to detect genome-wide significant associations with an odds ratio (OR) of 1.2 involving common variants with a minor allele frequency (MAF) of 21%. For rare single-nucleotide polymorphisms (SNPs) (MAF, 1%), the power surpassed 80% for an OR of 1.9.

## RESULTS

### Genome-wide association analyses

We recruited patients with either MS or clinically isolated syndrome (CIS) from MS centers throughout Germany and combined them with controls from several German population–based cohorts (Table 1). After quality control (QC), the data set DE1 consisted of 3934 cases and 8455 controls (control/case ratio, 2.15; table S1). We also compiled a second data set, called DE2, based on an independent group of German cases previously used in the IMSGC/WTCCC2 (International Multiple Sclerosis Genetics Consortium/Wellcome Trust Case Control Consortium 2) MS study (Table 1) (*5*), and additional German controls, mostly from population-based cohorts. The data set DE2 contained 954 cases and 1940 controls after QC (control/case ratio, 2.03; table S2). We observed only moderate population substructure within these data sets (figs. S1 and S2), confirming previous genetic analyses of the German population (*15*).

**Table 1:** Clinical characteristics of German MS cases. PPMS, primary progressive MS (as opposed to bout-onset MS).

	**Cohort DE1**	**Cohort DE2**
Number of cases	3934	954
Age [mean (range)]	39 (13–79)	40 (17–82)
Female [*n* (%)]	2723 (69.2)	695 (72.9)
Male [*n* (%)]	1211 (30.8)	259 (27.1)
PPMS [*n* (%)]	105 (2.7)	63 (6.6)

Both data sets were imputed separately to the 1000 Genomes Phase 1 reference panel using SHAPEIT2 and IMPUTE2 (*16*–*18*). The resulting data sets contained more than 8 million high-quality variants with MAFs of at least 1% each. We separately conducted genome-wide association studies (GWAS) on both data sets using sex and the first eight multidimensional scaling (MDS) components of the genetic similarity matrix (GSM) as covariates, to control for any remaining population substructure. After assuring that the median genomic inflation of the two GWAS was in the expected range (table S3), results were combined using a fixed-effects pooled analysis. In this pooled analysis, the genomic inflation λ_1000,1000_ outside the extended MHC region was 1.017 (table S3) (*19*).

### Associations within the MHC region

The variant showing the strongest association in the pooled analysis of DE1 and DE2, rs3104373 [OR, 2.90; confidence interval (CI), 2.72 to 3.09; *P* = 1.3 × 10^−234^], lies within the MHC region between the genes *HLA-DRB1* and *HLA-DQA1*. This SNP is in strong linkage disequilibrium (LD) with the *HLA* allele DRB1*15:01 (*r*^2^ = 0.99) and thus corresponds to the established major MS risk locus (*1*, *2*). To confirm this finding, we imputed classical *HLA* alleles from our genotyping data (*20*). After QC, we obtained high-quality imputed alleles for a total of 3966 cases and 8329 controls from DE1 and DE2 (median accuracy, 96.1%; median call rate, 97.4%). Using stepwise conditional logistic regression (*1*, *2*, *5*), seven *HLA* alleles (Table 2) reached genome-wide significance (that is, *P* < 5 × 10^−8^). As expected, the most significantly associated allele was DRB1*15:01 (OR, 2.85; CI, 2.66 to 3.06; *P* = 1.0 × 10^−191^). All seven alleles have been described as associated with MS in a recent detailed analysis of the MHC region (*2*).

**Table 2:** Genome-wide significant *HLA* alleles. Alleles are in order of stepwise logistic regression. For each row, alleles from the rows above have been used as covariates in the model. AF (allele frequency of controls in %) is calculated from a joint set of DE1 and DE2. ORs and *P* values are from a fixed-effects pooled analysis of DE1 and DE2.

***HLA* allele**	**AF**	**OR (95% CI)**	***P***	***HLA* alleles in LD (*r*^2^ > 0.9)**
DRB1*15:01	14.8	2.85 (2.66–3.06)	1.03 × 10^−191^	DQB1*06:02
A*02:01	28.6	0.68 (0.64–0.73)	3.68 × 10^−29^	
B*38:01	2.0	0.36 (0.27–0.49)	2.09 × 10^−11^	
DRB1*13:03	1.5	1.96 (1.60–2.40)	6.42 × 10^−11^	
DPB1*03:01	10.3	1.33 (1.22–1.46)	4.35 × 10^−10^	
DRB1*03:01	12.2	1.29 (1.18–1.40)	1.85 × 10^−8^	DQA1*05:01, DQB1*02:01
DRB1*08:01	3.0	1.63 (1.39–1.91)	2.36 × 10^−9^	DQA1*04:01, DQB1*04:02

Previous analyses of the MHC region have also identified associations between *HLA* alleles and age at onset of the disease, mainly with DRB1*15:01 (*2*, *5*). We confirmed this finding in a subset of patients from our data set DE1. Age at onset was known for 1519 patients; for 1196 of them, imputed *HLA* alleles were available. Because the age at onset was not normally distributed, rank-based inverse normal transformation was applied. The *HLA* allele most strongly associated with transformed age at onset was DRB1*15:01 (effect size, −0.21; *P* = 7.6 × 10^−6^). When conducting a genome-wide analysis of transformed age at onset in all 1519 patients, no variant passed the threshold for genome-wide significance (fig. S3, A and B). The most strongly associated SNP was rs4959027 (effect size, −0.20; *P* = 1.5 × 10^−7^; fig. S3, C and D), which is in LD with DRB1*15:01 (*r*^2^ = 0.72). After conditioning for DRB1*15:01 in the subset of cases with both age at onset and imputed *HLA* alleles available, the *P* value of rs4959027 was increased from 1.1 × 10^−6^ to 4.8 × 10^−2^. We conclude that our findings for the MHC region are very well in line with previous studies and concentrated further analyses on associations with case/control status outside this region.

### Associations outside the MHC region

**Fig. 1:**
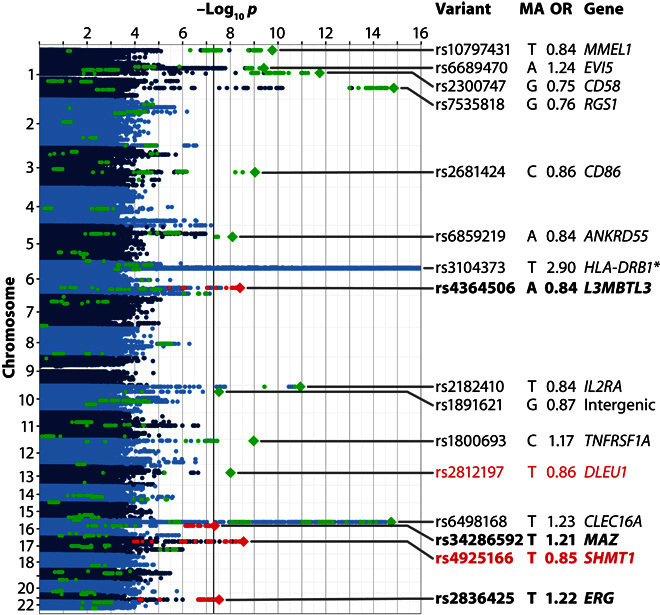
Genome-wide representation of MS associations in the pooled analysis of German data sets. Manhattan plot showing strength of evidence for association (*P* value). Each variant is shown as a dot, with alternating shades of blue according to chromosome. Green dots represent established MS-associated variants and their proxies, as listed by Sawcer *et al.* (*3*) (except for rs2812197, which was not covered by that review). Top variants at the 15 non-MHC loci associated at the genome-wide significance threshold in our study are shown as diamonds. Novel variants showing genome-wide significance are plotted as red diamonds; their names are shown in bold font. Variants in high LD (*r*^2^ ≥ 0.7) with these novel variants are shown as red dots. Variants replicating in the Sardinian cohort are shown in red font. MA, minor allele. The OR is relative to the MA. Gene names for known loci are indicated as listed by Sawcer *et al.* (*3*). The plot is truncated at −log_10_*p* = 16 for better visibility; all truncated variants map to the MHC region. The lowest *P* value (rs3104373, *) was 1.3 × 10^−234^.

Variants at 15 loci outside the MHC region showed genome-wide significance (Fig. 1, figs. S4 and S5, Table 3, and table S4). Ten of these loci have already been established in previous large MS GWAS (*3*, *4*, *6*). One more locus, *DLEU1* (*deleted in lymphocytic leukemia 1*), was only recently confirmed to be associated with MS in a candidate gene study (*21*). The remaining four signals are thus novel candidates for MS susceptibility loci. The lead variants at all 15 non-MHC loci showed *P* < 5 × 10^−6^ in DE1 and lower *P* < 5 × 10^−8^ in the pooled analysis of DE1 and DE2 and have thus replicated in DE2. We could not detect any significant interaction among the 15 top non-MHC variants or between them and SNP rs3104373 within the MHC region.

**Table 3:** Genome-wide significant loci outside the MHC region and the top variant within the MHC region. Bold font in the left half of the table indicates novel loci, whereas bold font in the right half indicates variants that replicated in Sardinians. All *P* values shown are two-sided. Gene names of known loci are as listed by Sawcer *et al.* (*3*). C, chromosome. For additional details, see table S4.

**Variant**	**C**	**MA**	**Gene**	**MAF DE**	**OR (CI) DE1 + DE2**	***P* DE1 + DE2**	***P* Sardinia**	**OR (CI) DE + Sardinia**	***P* DE + Sardinia**
rs10797431	1	T	*MMEL1*	34.1	0.84 (0.80–0.89)	1.81 × 10^−10^			
rs6689470	1	A	*EVI5*	14.2	1.24 (1.16–1.33)	3.93 × 10^−10^			
rs2300747	1	G	*CD58*	12.4	0.75 (0.69–0.81)	1.74 × 10^−12^			
rs7535818	1	G	*RGS1*	19.2	0.76 (0.71–0.82)	1.51 × 10^−15^			
rs2681424	3	C	*CD86*	49.7	0.86 (0.82–0.90)	9.51 × 10^−10^			
rs6859219	5	A	*ANKRD55*	22.2	0.84 (0.79–0.89)	8.06 × 10^−9^			
rs3104373	6	T	*HLA-DRB1*	13.6	2.90 (2.72–3.09)	1.34 × 10^−234^			
**rs4364506**	**6**	**A**	***L3MBTL3***	**26.4**	**0.84 (0.80–0.89)**	**4.06 × 10^−9^**	0.83	0.89 (0.85–0.93)	1.99 × 10^−6^
rs2182410	10	T	*IL2RA*	38.1	0.84 (0.79–0.88)	1.15 × 10^−11^			
rs1891621	10	G	Intergenic	46.7	0.87 (0.83–0.91)	2.94 × 10^−8^			
rs1800693	12	C	*TNFRSF1A*	42.1	1.17 (1.11–1.23)	1.06 × 10^−9^			
rs2812197	13	T	*DLEU1*	38.4	0.86 (0.82–0.91)	9.95 × 10^−9^	**6.86 × 10^−3^**	**0.87 (0.83–0.91)**	**2.83 × 10^−10^**
rs6498168	16	T	*CLEC16A*	35.5	1.23 (1.17–1.29)	1.98 × 10^−15^			
**rs34286592**	**16**	**T**	***MAZ***	**14.2**	**1.21 (1.13–1.30)**	**4.58 × 10^−8^**	0.44	1.16 (1.09–1.23)	4.79 × 10^-7^
**rs4925166**	**17**	**T**	***SHMT1***	**34.5**	**0.85 (0.81–0.90)**	**2.69 × 10^−9^**	**5.63 × 10^−4^**	**0.86 (0.82–0.90)**	**7.40 × 10^−12^**
**rs2836425**	**21**	**T**	***ERG***	**12.7**	**1.22 (1.14–1.31)**	**2.84 × 10^−8^**	0.35	1.18 (1.11–1.25)	1.54 × 10^−7^

**Fig. 2:**
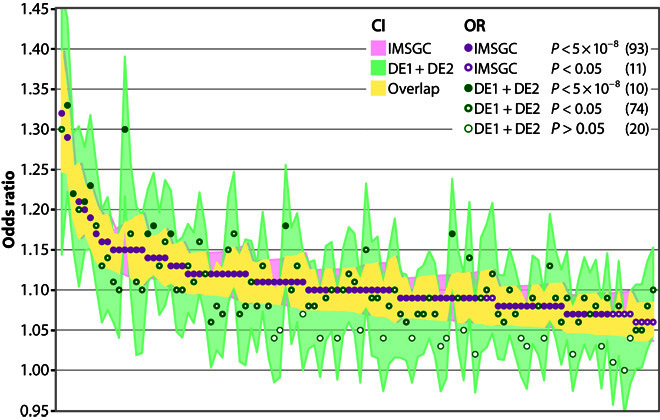
Comparison of results from the pooled analysis of Germans to associations found in an IMSGC study. One hundred and four of the 108 variants showing genome-wide significant or suggestive associations with MS in the study published by the IMSGC in 2013 (*4*) were present in the pooled results of DE1 and DE2. All 104 variants showed the same direction of effect (*P* = 5 × 10^−32^, binomial sign test). Fifty-eight variants had lower ORs and 35 higher ORs compared to the published data set. *P* value–based categories labeled with different dots represent exclusive bins that add up to 104.

For validation of our findings, we compared our results to the largest study on MS genetic susceptibility published to date (Fig. 2) (*4*). Of the 108 non-MHC variants showing genome-wide significant or suggestive associations with MS in the published study, 104 variants were present in our data and could be analyzed. All of them showed the same direction of effect (*P* = 5 × 10^−32^, binomial sign test; CI, 0.97 to 1.00), 84 with nominal (*P* < 0.05) and 10 with genome-wide significance (*P* < 5 × 10^−8^). Fifty-eight of the variants had lower ORs and 35 had higher ORs in our data than in the published data set (*4*). It was expected to observe more signals with lower ORs than previously reported due to regression toward the mean.

Next, we examined the four novel loci and *DLEU1* not found at genome-wide significance in a GWAS before in more detail. We investigated whether the five lead variants at these loci are significantly associated with MS in our German cohort only or whether they replicate in Sardinians, a genetically distinct population with low genetic heterogeneity. This independent Sardinian cohort consisted of 2903 cases (69.2% female, 1.2% PPMS) and 3323 controls (control/case ratio, 1.15) (*22*–*24*). Two of the variants (rs2812197 and rs4925166) replicated with *P* < 0.01 in the Sardinian data set; two more (rs34286592 and rs2836425) showed the same direction of effect but did not reach nominal significance (Table 3, table S4, and fig. S6).

### *SHMT1* as a novel MS susceptibility gene

**Fig. 3:**
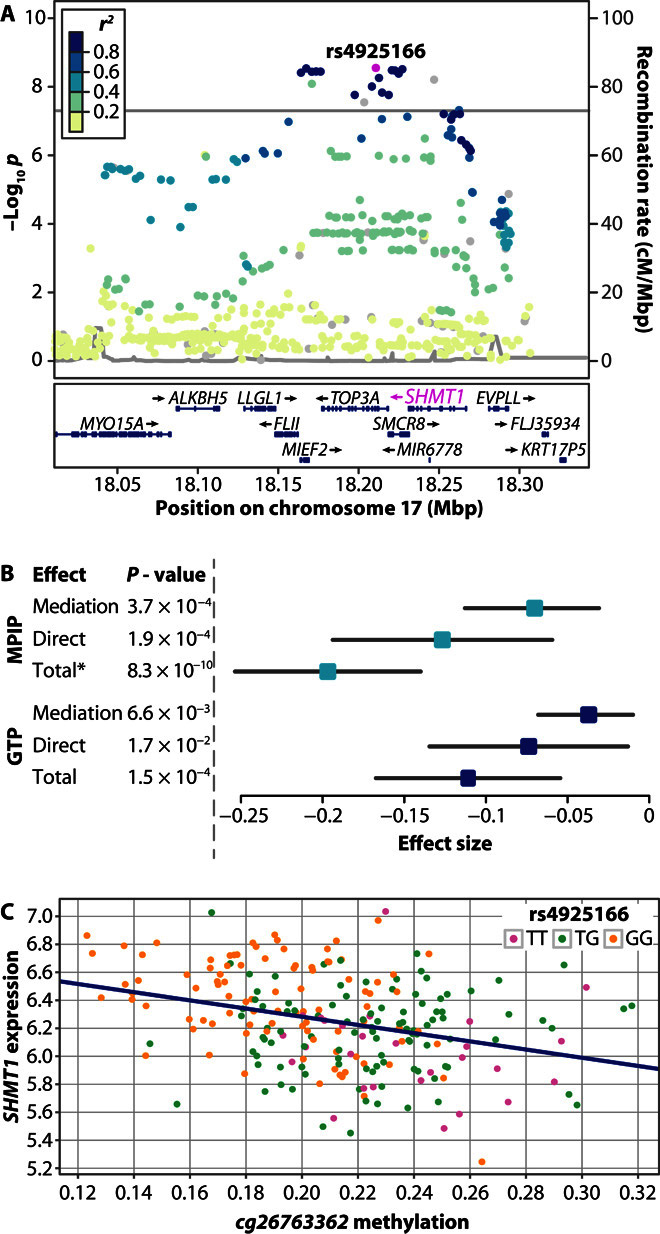
Fine-mapping analysis results of locus rs4925166. (**A**) Regional plot for the rs4925166/*SHMT1* locus. Color of dots indicates LD with the lead variant (rs4925166; pink). Gray dots represent signals with missing *r*^2^ values. cM, centimorgan. (**B**) Mediation analysis results in MPIP/GTP controls. Mediation effect: rs4925166→CpG cg26763362→*SHMT1* expression. Direct effect: rs4925166→*SHMT1* expression. Data have been calculated using the R package mediation (*30*), except for total effect (*), which was calculated by linear regression. Results were obtained using 1 million simulations. Effects and *P* values shown here differ from Table 5, as a lower number of samples contained both expression and methylation data than expression data alone. (**C**) Relationship between *cg26763362* methylation, *SHMT1* expression, and rs4925166 genotype in MPIP controls.

The association of rs4925166 constituted the strongest signal among the novel variants. It showed an OR of 0.85 (CI, 0.81 to 0.90) and a *P* value of 2.7 × 10^−9^ in the pooled analysis of German data sets (Table 3). This variant replicated in the Sardinian cohort with a joint *P* value of 7.4 × 10^−12^ (fig. S6D). SNP rs4925166 is located on chromosome 17 in an intron of the gene *TOP3A*, coding for the DNA topoisomerase IIIα. However, strongly associated SNPs in this genomic region spread over several neighboring genes (Fig. 3A). We therefore conducted an expression quantitative trait locus (eQTL) analysis using a subset of 242 patients from data set DE1 to functionally link variants to nearby genes. We examined transcripts within a cis window of 1 million base pairs upstream and downstream of the lead variant for an association of blood gene expression levels with allele configuration (table S5). The variant rs4925166 and proxy SNPs (*r*^2^ > 0.7) were found to be part of a strong eQTL with the gene *SHMT1* in DE1 samples [false discovery rate (FDR), 2.99 × 10^−10^; Table 4 and fig. S7, A to C]. This eQTL was replicated in two independent control data sets [Max Planck Institute of Psychiatry (MPIP) data (*25*) and Grady Trauma Project (GTP) (*26*–*28*)] and in the publicly available GTEx eQTL database (*29*) (Table 4).

**Table 4:** eQTL and mQTL analysis for rs4925166. Direction of effect is relative to the minor allele T. Note that the effect sizes cannot be directly compared because normalization methods and covariates partly differ between studies. Additional eQTLs and mQTLs are described in the Supplementary Materials. Because only the single eQTL rs4925166/*SHMT1* was examined in GTEx data, no FDR is indicated here. NA, not applicable.

***Expression***				
**Data set**	**Transcript**	**Effect**	***P***	**FDR**
DE1	*SHMT1*	0.36	4.42 × 10^−13^	2.99 × 10^−10^
MPIP	*SHMT1*	0.19	4.26 × 10^−12^	1.28 × 10^−11^
GTP	*SHMT1*	0.11	3.12 × 10^−4^	1.25 × 10^−3^
GTEx	*SHMT1*	0.56	09.2 × 10^−28^	NA
*Methylation*				
**Data set**	**CpG**	**Effect**	***P***	**FDR**
MPIP	*cg26763362*	−0.03	3.21 × 10^−20^	5.04 × 10^−18^
GTP	*cg26763362*	−0.03	1.98 × 10^−14^	1.58 × 10^−13^

To investigate how rs4925166 influences the expression of *SHMT1*, we conducted an association analysis of the SNP with DNA methylation levels in blood. DNA methylation is an important epigenetic mechanism for regulation of gene expression. We tested the association between rs4925166 and DNA methylation levels at CpG sites in the two non-MS data sets MPIP and GTP. Methylation levels at 157 CpG sites that mapped to *SHMT1* were examined for an association with genotype. We observed eight significant (FDR <0.05) methylation QTLs (mQTLs) between rs4925166 and CpGs in *SHMT1* within the MPIP data set. Three of these associations could be replicated in the GTP data set (table S6 and fig. S7, D and E).

We wondered whether the CpG site showing the strongest association with rs4925166 (cg26763362) could fully explain the observed association between the SNP and *SHMT1* expression (causal direction: rs4925166→cg26763362→*SHMT1* expression) using mediation analysis (Table 4, tables S7 and S8, Fig. 3) (*30*). We observed partial mediation of the effect of rs4925166 on *SHMT1* expression by DNA methylation status of CpG site cg26763362. The association pattern indicates that an additional factor influences the relationship between the SNP, the CpG, and the gene expression (see the Supplementary Materials). Thus, we conclude that the genotype of rs4925166 affects the expression of *SHMT1* in a complex fashion, partially involving rs4925166-dependent DNA methylation.

**Table 5:** Fine-mapping of the *DLEU1* locus. MAF (controls in %) and *r*^2^ (with rs2812197) are calculated from a joint set of DE1 and DE2, ORs and *P* values from the pooled analysis of DE1 and DE2. Second and third *P* value columns are from conditional analysis.

**Variant**	**MAF**	**OR (CI)**	***P***	***P* (rs2812197)**	***P* (rs9591325)**	***r*^2^**	**Reference**
rs2812197	38.4	0.86 (0.82–0.91)	9.95 × 10^−9^		4.79 × 10^−5^	1.00	
rs806321	48.5	0.89 (0.85–0.94)	6.36 × 10^−6^	0.81	2.02 × 10^−3^	0.66	(*5*)
rs806349	46.0	1.10 (1.04–1.15)	2.73 × 10^−4^	0.99	0.019	0.41	(*4*, *21*)
rs9591325	8.1	0.78 (0.70–0.85)	2.26 × 10^−7^	9.13 × 10^−4^		0.14	
rs9596270	8.1	0.78 (0.71–0.86)	4.45 × 10^−7^	1.49 × 10^−3^	0.27	0.14 (0.99*)	(*6*)

*r^2^ with rs9591325.

### Additional novel candidate loci associated with MS

Three loci showed genome-wide significance in the pooled analysis of German data sets DE1 and DE2 but not in Sardinians (Table 3). The strongest association, SNP rs4364506, was found on chromosome 6 and is located in an intron of the gene coding for the transcriptional regulator L3MBTL3 [Lethal(3)malignant brain tumor–like protein 3; fig. S5G). SNP rs2836425 on chromosome 21 constituted the second strongest signal identified in Germans only. This variant maps to an intron of the gene *ERG*, coding for a transcription factor (fig. S5P). The third SNP rs34286592 is located in an intron of the gene *MAZ* on chromosome 16, coding for the transcription factor MYC-associated zinc finger protein (fig. S5N). It maps to binding sites for transcription factors (fig. S8G).

When conditioning for the lead variants at the four newly identified MS-associated loci, no evidence for secondary signals was found. Thus, the lead variants also constitute the most likely causal variants. These variants all map to introns of genes. This makes a functional link between each variant and the gene it is located in probable. To further explore the functional connections between SNPs and genes, we conducted an eQTL analysis of the 15 loci showing genome-wide significant associations. We thereby identified four cis-eQTLs with FDR <0.05 in MS cases (table S5). In addition to the eQTL of rs4925166 and *SHMT1* already described above, three more significant eQTLs involved variants at two previously known MS susceptibility loci and three transcripts of the genes *MMEL1* and *ANKRD55*.

### Fine-mapping of *DLEU1*

Three variants located on chromosome 13 (rs806321, rs9596270, and rs806349), all intronic within the gene for the long noncoding RNA *DLEU1*, have been described previously as associated with MS in three large studies (*4*–*6*), yet the variants did not show genome-wide significance in any of them. The association of rs806349 has recently been confirmed in a candidate-driven follow-up analysis of suggestive MS associations (*21*). However, the variant rs806349 reached a *P* value of only 2.7 × 10^−4^ in our analysis (Table 5). Instead, a different SNP (rs2812197) in weak LD with rs806349 (*r*^2^ = 0.4) showed genome-wide significance in the pooled analysis of DE1 and DE2 and also replicated in Sardinians (Tables 3 and 5, and fig. S5K). The association of previously described rs806349 is completely dependent on the more strongly associated rs2812197 (Table 5). Thus, it is unlikely that rs806349 is the causal SNP at this locus. The same is true for rs806321 (*5*), which is not independent of rs2812197 either (Table 5).

The *DLEU1* locus contains evidence for a second signal, rs9591325 (Table 5 and fig. S5L), in poor LD with rs2812197, but in high LD with rs9596270, which was identified by Patsopoulos *et al.* (*6*) as a suggestive MS-associated variant. The two signals were partially independent of each other (Table 5). SNP rs9591325 is located in a clearly functional region with binding sites for many transcription factors, which is not the case for the other four variants (fig. S8, B to F). Although rs2812197 shows the overall strongest association at *DLEU1*, the functional data indicate that rs9591325 might be either the actual or a second causal variant. Additional studies with larger sample sizes are required to fully answer this question.

## DISCUSSION

The present study constitutes the largest GWAS on MS conducted in a single population to date. By pooled analysis of 3934 cases in data set DE1 and 954 cases in data set DE2, we identified strong associations in the MHC region with a *P* value of up to 1.3 × 10^−234^. In addition, 15 loci outside the MHC region were associated at a genome-wide significant level (Fig. 1 and Table 3). Associations in the MHC region were examined using imputed *HLA* alleles. Stepwise conditional logistic regression identified DRB1*15:01 and six more associated *HLA* alleles (Table 2), in line with results from previous studies (*2*). All genome-wide significant and suggestive non-MHC MS susceptibility variants published by the IMSGC in 2013 (*4*) and present in our data (*n* = 104) were replicated regarding direction of effects in our samples (*P* = 5 × 10^−32^; Fig. 2).

Four of the 15 non-MHC loci have not been found to be associated with MS in previous studies. One more locus, *DLEU1*, did not reach genome-wide significance in previous GWAS but has recently been confirmed as MS-associated in a candidate SNP study (*21*). The lead variants at *DLEU1* and at the novel locus *SHMT1* replicated in an independent Sardinian cohort containing 2903 cases (Table 3 and fig. S6). Variants at the other three novel loci did not reach nominal significance in Sardinians, yet two of them showed the same direction of effect. Because of their consistency and replication within the German cohorts, these three associations can nevertheless be considered as plausible. As the Sardinian population is genetically distinct from Germans, future studies are required to replicate these findings in other cohorts.

Previous genetic analyses of MS susceptibility have indicated immune system–related processes as relevant for the development of MS (*4*). Functions of known MS susceptibility genes have been mapped to KEGG (Kyoto Encyclopedia of Genes and Genomes) pathways Janus kinase/signal transducers and activators of transcription (JAK/STAT) signaling, acute myeloid leukemia (AML), and T cell receptor signaling (*7*). Accordingly, MS-associated genes are predominantly expressed in immune cells (*7*, *8*). The five genes examined in detail in our study (*L3MBTL3*, *DLEU1*, *MAZ*, *ERG*, and *SHMT1*) are associated with regulatory mechanisms in immune cells as well.

The gene *L3MBTL3* encodes a Polycomb group protein that maintains the transcriptionally repressive state of genes (*31*) and is frequently deleted in several forms of acute leukemia, including AML (*32*). Genes associated with AML constitute one of the most significant pathway categories linked to MS susceptibility variants (*7*). The murine ortholog of *L3MBTL3*, *MBT-1*, has been found to regulate maturation of myeloid progenitor cells (*33*). The regulatory long noncoding RNA *DLEU1* is often deleted in cases of B cell chronic lymphocytic leukemia and mantle cell leukemia (*34*). This locus regulates the expression of *NF-*κ*B* (*35*), a transcription factor implicated in MS pathology (*4*, *36*, *37*). MAZ is an inflammation-responsive transcription factor (*38*) up-regulated during chronic myeloid leukemia (*39*). It binds to the promoter of the gene *MYC*, which is associated with MS (*5*). The transcription factor ERG is important for hematopoiesis (*40*), and the expression of this oncogene is associated with both AML and acute T cell lymphoblastic leukemia (*41*). ERG regulates the expression of MS-associated *NF-*κ*B* (*42*), as *DLEU1* does. Finally, SHMT1 is a serine hydroxymethyltransferase acting in the folate cycle. It catalyzes the transfer of a carbon unit subsequently used for synthesis of both nucleotides and methionine. SHMT1 is thus an essential component in the metabolism of the substrate *S*-adenosylmethionine (SAM), the major methyl group donor during both protein and DNA methylation (*43*, *44*). By this effect on regulation of gene expression, one-carbon metabolism plays an important role in oncogenesis. Lack of *SHMT1* function is, among other effects, associated with acute lymphocytic leukemia (*44*–*46*). Thus, each of the five genes is involved in regulatory processes of the immune system.

Although a clearer picture has already emerged regarding the cell types and broad pathways relevant for the etiology of MS (*3*, *7*), little is still known about the mechanisms by which risk genes act. Analysis of the known functions of the five genes examined in this study revealed that four of them regulate transcription, especially of immune-related genes. Moreover, indirect evidence suggests that they could all be linked either directly or indirectly to epigenetic regulatory mechanisms: L3MBTL3 recognizes epigenetic histone lysine methylation (*31*) and ERG interacts with ESET, a histone H3–specific methyltransferase (*47*). The best known regulatory target of the transcription factor MAZ is *MYC* (*48*), a regulator of epigenetic chromatin state that is associated with MS (*5*, *49*). *DLEU1* is strictly regulated by DNA methylation at its promoter region (*35*). Finally, SHMT1 is essential for maintaining methylation homeostasis in the cell by catalyzing an important reaction in the generation of the methyl donor substrate SAM. Accordingly, the establishment of SHMT1 as an MS risk factor further puts epigenetic regulation by methylation in the focus of MS susceptibility.

In recent years, several studies have addressed the role of DNA methylation in the etiology and progression of MS. Methylation differences between MS cases and healthy controls have been analyzed in small, cross-sectional studies. Despite negative results in CD4^+^ cells (*12*, *50*), Bos and colleagues (*12*) recently observed significant differences in overall DNA methylation levels in CD8^+^ T cells. Another study demonstrated differentially methylated and expressed genes in brain tissue of MS patients compared to controls (*14*). Furthermore, differential methylation of the major risk locus *HLA-DRB1* was observed in MS patients (*51*). Several groups have found either hypermethylation or hypomethylation of specific genes to be associated with inflammation or demyelination in MS patients (*11*).

In summary, these studies argue in favor of DNA methylation being relevant for the development of MS. By finding novel risk genes with potential roles in epigenetic regulation, our study adds further indication that epigenetic mechanisms might be important for MS susceptibility. A disturbed homeostasis of methyl donors, caused by an altered expression of *SHMT1*, is likely to have an impact on the disease. As epigenetic mechanisms constitute a major route for environmental risk factors to influence expression of disease-associated genes (*11*), regulation of DNA and protein methylation is an interface where genetic and environmental risk factors for MS might intersect. Detailed analyses of DNA methylation patterns and their interaction with MS susceptibility genes in larger cohorts and among different cell populations and tissues are now required to better understand the role of epigenetic mechanisms in MS.

## MATERIALS AND METHODS

### Study samples

Two cohorts of cases, referred to as DE1 and DE2, were analyzed. Both data sets included patients with CIS, bout-onset MS, and PPMS. For cohort DE1, 4503 cases were recruited across multiple sites in Germany (for details, see the Supplementary Materials). For cohort DE2, 1002 cases were recruited across multiple sites in Germany (see the Supplementary Materials). The latter cohort was used in a previous publication (*5*). Controls for these cohorts were obtained from several population-based cohorts across Germany to match the different geographical regions where cases were recruited: KORA from the southeastern Germany region of Augsburg (*52*, *53*), HNR from central western Germany (*54*), SHIP from the northeastern region of West Pomerania (*55*), DOGS from Dortmund in central western Germany (*56*), and FoCus (*57*) and PopGen (*58*) from Kiel, northern Germany. In addition, controls from two studies on depression conducted in southeastern Germany were included (*59*, *60*). For a more detailed description of control cohorts, see the Supplementary Materials. All responsible ethics committees provided positive votes for the individual studies. All study participants gave written informed consent. In case of minors, parental informed consent was obtained.

### Genotyping and QC

Samples of cohort DE1 were genotyped using the Illumina HumanOmniExpress-24 v1.0 or v1.1 BeadChips. Samples of cohort DE2 were genotyped using the Illumina Human 660-Quad platform. For both cohorts, identical, stringent QC was conducted on samples and variants. QC steps on samples included removal of individuals with genotyping rate <2%, cryptic relatives (relatedness ≥1/16), and genetic population outliers. QC steps on variants included removal of variants with call rate <2% and MAF <1%. For a full description of QC, see the Supplementary Materials. Each set of cohorts was combined with controls genotyped on similar arrays, producing case/control data sets DE1 and DE2. QC was repeated on the merged data sets, leading to final figures of 3934 cases and 8455 controls for DE1 (table S1), as well as 954 cases and 1940 controls for DE2 (table S2).

### Imputation

Prephasing (haplotype estimation) of genotype data was conducted using SHAPEIT2, followed by imputation using IMPUTE2 in 5–megabase pair (Mbp) chunks (*16*–*18*). The 1000 Genomes Phase 1 June 2014 release was used as a reference panel. Imputed variants were filtered for MAF (≥1%), INFO metric (≥0.8), and HWE (*P* ≥ 10^−6^). For additional details, see the Supplementary Materials.

*HLA* alleles were separately imputed from genotyping data for DE1 and DE2 using HIBAG v1.6.0 (*20*). Alleles with a posterior probability >0.5 were converted to hard calls. Results were validated using *HLA* typing of 442 patients from DE1 (see the Supplementary Materials).

### Statistical analyses of genotype data

GWAS was conducted on data sets DE1 and DE2 using PLINK2 v1.90b3s (*61*). Sex and the first eight MDS components were used as covariates in logistic regression. Data sets were combined using a fixed-effects model in METASOFT (*62*). For maximum precision, logistic regression and meta-analysis of lead SNPs were repeated in R v3.2.3 using package meta v4.3.2. All follow-up analyses (for example, conditional and interaction analyses) were conducted in R. Locus-specific Manhattan plots were generated using LocusZoom with European samples of the 1000 Genomes March 2012 reference panel on the hg19 build (*63*). For analysis of *HLA* alleles, stepwise logistic regression was conducted in R as previously described (*1*, *2*, *5*).

### Gene expression and methylation data

For a subset of 242, mostly treatment-naïve patients from data set DE1 (73 male and 169 female) whole-blood RNA was collected using Tempus Blood RNA Tubes (Applied Biosystems). RNA was hybridized to Illumina HT-12 v4 Expression BeadChips (Illumina) and further processed as described in the Supplementary Materials. In summary, QC was conducted in R 3.2.1 using the packages beadarray and lumi (*64*, *65*). Probes were transformed and normalized through variance stabilization and normalization (*66*). Probes, which showed a detection *P* < 0.05 in more than 10% of the samples, which could not be mapped to a known transcript, or which were identified as cross-hybridizing by the Re-Annotator pipeline (*67*), were removed. This left 20,302 transcripts from 242 samples. Technical batch effects were identified by inspecting the association of the first two principal components of expression levels with amplification round, amplification plate, and amplification plate column and row, as well as expression chip. The data were then adjusted using ComBat (*68*). Gene expression and methylation data of the two control cohorts MPIP and GTP were published and described previously and are summarized in the Supplementary Materials (*25*–*28*).

### Statistical analysis of gene expression and methylation data

For each of the 15 genome-wide significant loci, all 429 transcripts beginning or ending within 1 Mbp upstream or downstream of a lead variant were determined. Associations between genotype and expression levels were examined in data set DE1 by linear regression using sex, age, and three MDS components as covariates. To account for multiple testing, *P* values were first corrected for the number of transcripts per cis window, followed by calculation of the FDR for the total number of variants tested. Replication of eQTLs with an FDR <0.05 in data set DE1 was conducted in control cohorts MPIP and GTP. For MPIP, the covariates sex, age, body mass index (BMI), disease status, and three MDS components were used in linear regression. For GTP, covariates were sex, age, and four MDS components. eQTLs were also looked up in the GTEx database (*29*). Here, only associations in whole blood were considered.

For analysis of the association of rs4925166 with DNA methylation at *SHMT1*, 210 CpG probes were identified in data set MPIP that mapped to *SHMT1*. After removing the quartile of probes showing the lowest variation in methylation status, 157 CpGs remained. Association of DNA methylation with imputed genotype was assessed by linear regression using sex, age, BMI, disease status, three MDS components, and estimated cell counts as covariates. The eight CpG probes showing an FDR <0.05 were replicated in data set GTP, using sex, age, four MDS components, and estimated cell counts as covariates. Mediation analysis was conducted as outlined in the Supplementary Materials, including nonparametric bootstrap for estimation of CIs and *P* values (*30*).

### Replication of the results in a Sardinian cohort

The replication case group consisted of 2903 unrelated Sardinian MS patients that were diagnosed and selected using the McDonald criteria (*22*–*24*). Only 35 of these patients were diagnosed with PPMS (1.2%). Two thousand ten (69.2%) cases were female, and 893 (30.8%) were male; the average age at onset was 32 years. The matching control group of healthy individuals is composed of 2880 unrelated adult volunteer blood donors from the same locations where the cases were collected, as well as 443 Affected Family BAsed pseudo-Controls (AFBACs) derived from 242 MS and 201 type 1 diabetes family trios (*23*). AFBAC allele and haplotype frequencies were constructed using the two alleles in each trio that are not transmitted from the parents to the affected child. These familial pseudo-controls are matched to the cases for ethnic origin and are thus robust to population stratification.

All individuals were genotyped using the Illumina ImmunoChip array. In addition, 2040 (962 cases and 1078 controls) were genotyped with the Illumina HumanOmniExpress array and 3917 (2111 cases and 1806 controls) with the Affymetrix 6.0 array. One hundred seventy-four individuals (170 case and 4 controls) were genotyped using both HumanOmniExpress and Affymetrix 6.0 (*22*). After QC, we used 883,557 SNPs as baseline for imputation (*17*) of 20.1 million untyped SNPs using a Sardinian-specific reference panel, including 3514 Sardinian individuals sequenced to an average coverage of 4.16-fold (*69*).

## Supplementary Material

http://advances.sciencemag.org/cgi/content/full/2/6/e1501678/DC1

## SUPPLEMENTARY MATERIALS

Supplementary material for this article is available at http://advances.sciencemag.org/cgi/content/full/2/6/1501678/DC1 Supplementary Results Supplementary Materials and Methods table S1. QC of data set DE1. table S2. QC of data set DE2. table S3. Genomic inflation. table S4. Genome-wide significant loci. table S5. eQTLs with FDR <0.05 in data set DE1. table S6. Replicated mQTLs of rs4925166 and CpG sites in *SHMT1*. table S7. Mediation analysis. table S8. Causal mediation analysis. fig. S1. Substructure analysis results in DE1. fig. S2. Substructure analysis results in DE2. fig. S3. GWAS with age at onset. fig. S4. Forest plots of all non-MHC top genome-wide significant variants. fig. S5. Locus-specific Manhattan plots. fig. S6. Forest plots of novel variants replicated in a Sardinian cohort. fig. S7. eQTL and mQTL analysis for rs4925166. fig. S8. Transcription factor binding sites. References (*70*–*75*)
