# Supplementary material for: Novel multiple sclerosis susceptibility loci implicated in epigenetic regulation
Source: Sci Adv. 2016 Jun 17;2(6):e1501678. doi: 10.1126/sciadv.1501678 (PMC4928990; doi:10.1126/sciadv.1501678)
Supplement: http://advances.sciencemag.org/cgi/content/full/2/6/e1501678/DC1 [file supp_2_6_e1501678__index.html]

Science Advances | Science Advances

## Supplementary Materials

**This PDF file includes:**

- Supplementary Results
- Supplementary Materials and Methods
- table S1. QC of data set DE1.
- table S2. QC of data set DE2.
- table S3. Genomic inflation.
- Legends for tables S4 and S5
- table S6. Replicated mQTLs of rs4925166 and CpG sites in *SHMT1*.
- table S7. Mediation analysis.
- table S8. Causal mediation analysis.
- fig. S1. Substructure analysis results in DE1.
- fig. S2. Substructure analysis results in DE2.
- fig. S3. GWAS with age at onset.
- fig. S4. Forest plots of all non-MHC top genome-wide significant variants.
- fig. S5. Locus-specific Manhattan plots.
- fig. S6. Forest plots of novel variants replicated in a Sardinian cohort.
- fig. S7. eQTL and mQTL analysis for rs4925166.
- fig. S8. Transcription factor binding sites.
- References (*70–75*)

Download PDF

**Other Supplementary Material for this manuscript includes the following:**

- table S4 (Microsoft Excel format). Genome-wide significant loci.
- table S5 (Microsoft Excel format). eQTLs with FDR <0.05 in data set DE1.

Download Tables S4 and S5

**Files in this Data Supplement:**

- Adobe PDF - 1501678\_SM.pdf
