# Supplementary material for: Novel multiple sclerosis susceptibility loci implicated in epigenetic regulation
Source: Sci Adv. 2016 Jun 17;2(6):e1501678. doi: 10.1126/sciadv.1501678 (PMC4928990; doi:10.1126/sciadv.1501678)
Supplement: http://advances.sciencemag.org/cgi/content/full/2/6/e1501678/DC1 [file 1501678_SM.pdf]

Science Advances

AAAS

advances.sciencemag.org/cgi/content/full/2/6/e1501678/DC1

Supplementary Materials for

Novel multiple sclerosis susceptibility loci implicated in epigenetic regulation

Till F. M. Andlauer, Dorothea Buck, Gisela Antony, Antonios Bayas, Lukas Bechmann, Achim Berthele, Andrew Chan, Christiane Gasperi, Ralf Gold, Christiane Graetz, Jürgen Haas, Michael Hecker, Carmen Infante-Duarte, Matthias Knop, Tania Kümpfel, Volker Limmroth, Ralf A. Linker, Verena Loleit, Felix Luessi, Sven G. Meuth, Mark Mühlau, Sandra Nischwitz, Friedemann Paul, Michael Pütz, Tobias Ruck, Anke Salmen, Martin Stangel, Jan-Patrick Stellmann, Klarissa H. Stürner, Björn Tackenberg, Florian Then Bergh, Hayrettin Tumani, Clemens Warnke, Frank Weber, Heinz Wiendl, Brigitte Wildemann, Uwe K. Zettl, Ulf Ziemann, Frauke Zipp, Janine Arloth, Peter Weber, Milena Radivojkov-Blagojevic, Markus O. Scheinhardt, Theresa Dankowski, Thomas Bettecken, Peter Lichtner, Darina Czamara, Tania Carrillo-Roa, Elisabeth B. Binder, Klaus Berger, Lars Bertram, Andre Franke, Christian Gieger, Stefan Herms, Georg Homuth, Marcus Ising, Karl-Heinz Jöckel, Tim Kacprowski, Stefan Kloiber, Matthias Laudes, Wolfgang Lieb, Christina M. Lill, Susanne Lucae, Thomas Meitinger, Susanne Moebus, Martina Müller-Nurasyid, Markus M. Nöthen, Astrid Petersmann, Rajesh Rawal, Ulf Schminke, Konstantin Strauch, Henry Völzke, Melanie Waldenberger, Jürgen Wellmann, Eleonora Porcu, Antonella Mulas, Maristella Pitzalis, Carlo Sidore, Ilenia Zara, Francesco Cucca, Magdalena Zoledziewska, Andreas Ziegler, Bernhard Hemmer, Bertram Müller-Myhsok

Published 17 June 2016, *Sci. Adv.* **2**, e1501678 (2016)
DOI: 10.1126/sciadv.1501678

This PDF file includes:

- Supplementary Results
- Supplementary Materials and Methods
- table S1. QC of data set DE1.
- table S2. QC of data set DE2.
- table S3. Genomic inflation.
- Legends for tables S4 and S5
- table S6. Replicated mQTLs of rs4925166 and CpG sites in *SHMT1*.
- table S7. Mediation analysis.
- table S8. Causal mediation analysis.
- fig. S1. Substructure analysis results in DE1.
- fig. S2. Substructure analysis results in DE2.
- fig. S3. GWAS with age at onset.
- fig. S4. Forest plots of all non-MHC top genome-wide significant variants.

- fig. S5. Locus-specific Manhattan plots.
- fig. S6. Forest plots of novel variants replicated in a Sardinian cohort.
- fig. S7. eQTL and mQTL analysis for rs4925166.
- fig. S8. Transcription factor binding sites.
- References (*70–75*)

Other Supplementary Material for this manuscript includes the following:
(available at advances.sciencemag.org/cgi/content/full/2/6/e1501678/DC1)

- table S4 (Microsoft Excel format). Genome-wide significant loci.
- table S5 (Microsoft Excel format). eQTLs with FDR <0.05 in data set DE1.

Samples

All responsible ethics committees have provided positive votes for the individual studies. All study participants gave written informed consent. In case of minors, parental informed consent was obtained in addition.

Case collection DE1

4,503 cases have been recruited, 3,934 of them were included after quality control (QC). The following institutions provided these cases:

- Cohort of the German Competence Network Multiple Sclerosis (KKNMS): 1,019 patients of this prospective study were included. Patients were diagnosed with a clinically isolated syndrome (CIS) or an early stage of bout onset MS and were recruited in different hospitals within Germany.
- Munich TUM: 1,595 samples from the TU Munich were included, 43 of which have been diagnosed with PPMS, the remaining patients with either CIS or bout onset MS. The samples can be stratified into three subcohorts, recruited from South-Eastern Germany, from central Germany, and throughout Germany.
- Munich MPI of Psychiatry: 261 samples from the MPIP were included, 11 of which have been diagnosed with PPMS, 1 with neuromyelitis optica (NO), 1 with transverse myelitis, and the remainder with either CIS or bout onset MS. The cohort was recruited at the outpatient clinic of the institute. The study was approved by the ethics committee of the Medical Faculty at the Ludwig Maximilians University, Munich.
- Münster: 250 samples recruited in North-Western Germany at the Department of Neurology, University of Münster were included, 7 of which have been diagnosed with PPMS, the remainder with bout onset MS.
- Mainz: 224 samples recruited at the Department of Neurology, University Medical Center of the Johannes Gutenberg University Mainz were included, 25 of which have been diagnosed with PPMS, the remainder with either CIS or bout onset MS. The inclusion of patients in this study was independent of the use of disease-modifying treatments. The study was approved by the ethics committee of the medical association of Rheinland-Pfalz with the approval ID 837.019.10(7028).
- Bochum: 144 samples recruited at different sites across Germany were included, 3 of which have been diagnosed with PPMS, 1 with NO, and the remainder with bout onset MS. All patients were treated with Mitoxantrone. Samples were obtained under a protocol approved by the local ethics committee (Ethik-Kommission der Ruhr-Universität Bochum, reg.-nr. 4319-12).
- Hamburg HETOMS: 105 samples from Northern Germany, recruited at the MS outpatient unit of the University Medical Center Hamburg-Eppendorf, were included, all diagnosed with either CIS or bout onset MS according to the McDonald Criteria 2005. The study was approved by the local ethics committee (Ethik-Kommission der Ärztekammer Hamburg).

- Berlin: 96 samples recruited at the Clinical and Experimental MS Research Center, Charité - Universitätsmedizin Berlin were included, all diagnosed with bout onset MS. The study was approved by the local ethics committee.
- Rostock: 80 samples recruited at the Department of Neurology, University of Rostock were included, 5 of which have been diagnosed with PPMS, the remainder with either CIS or bout onset MS.
- Heidelberg: 59 samples recruited in South-Western Germany at the Department of Neurology, University of Heidelberg were included, all of which have been diagnosed with either CIS or bout onset MS.
- Marburg: 56 samples recruited at an outpatient clinic at the University of Marburg were included, 2 of which have been diagnosed with PPMS, the remainder with bout onset MS.
- Leipzig: 45 samples recruited at the Department of Neurology, University of Leipzig were included, 3 of which have been diagnosed with PPMS, the remainder with either CIS or bout onset MS. The study was approved by the ethics committee of the University of Leipzig.

Case collection DE2

This cohort has been described and published previously (*5*). It consists of 1,002 patients from two different cohorts, one from central Germany and the other from across multiple sites of Germany. 954 of them were included after QC, 63 of these cases have been diagnosed with PPMS, the remainder with bout onset MS.

Control collection DE1

Population-based cohorts used as controls were KORA from the South-Eastern German region of Augsburg (KORA-S3F3, 3,566 samples were included) (*52*, *53*), HNR from central Western Germany (2,451 samples included) (*54*), SHIP from the North-Eastern region West Pomerania (SHIP-Trend, 937 samples included) (*55*), DOGS from Dortmund in central Western Germany (895 samples included) (*56*), and FoCus from Kiel in Northern Germany (606 samples included) (*57*).

Control collection DE2

Population-based cohorts used as controls were popgen from Kiel in Northern Germany (624 samples included) (*58*) and KORA from the South-Eastern German region of Augsburg (KORA-S4F4, 414 samples were included) (*52*, *53*). In addition, controls of two studies on depression were used, recruited in South-Eastern Germany, GSK-Munich (818 samples were included) (*59*) and MARS (84 samples were included) (*60*).

Quality control

Quality control of genotyped data was conducted in PLINK 1.90b3s (*61*).

Quality control of imputed probabilities was conducted in QCTOOL 1.4 (http://www.well.ox.ac.uk/~gav/qctool/).

Samples were filtered according to the following criteria:

Individual genotyping rate	≥ 98 %
Cryptic relatedness (PI-HAT)	< 1/16
Outlier: distance in first two MDS components from mean	< 5 SD†
Deviation of autosomal heterozygosity from mean	< 4 SD
Heterozygosity on X	> -0.2

† Where required, a more stringent threshold was selected for a second round of outlier removal. This threshold was based on individual MDS plots.

Variants were filtered according to the following criteria:

Variant call rate	≥ 98 %
Minor allele frequency	≥ 1 %
HWE test *p*-value	$\geq 10^{-6}$ †
Variants on non-autosomal chromosomes	
Ambivalent SNPs (A/T and G/C)	
Variants not present in the 1000 genomes phase I reference panel	
IMPUTE2 info metric	≥ 0.8

† For cases, variants on chromosome 6 were removed before imputation only if HWE $p < 10^{-50}$, after imputation $p < 10^{-6}$ was used for all variants.

Application of these QC criteria led to the following data sets:

Cohort	Origin	Samples pre QC	Samples post QC	Male [%]	Chip	Variants pre QC	Variants post QC
DE1 cases	Germany (G)	4,503	3,934	30.8	OE	729,801	608,315
KORA-S3F3	SE G	4,086	3,566	50.0	O2.5	2,380,310	586,472
HNR-1	W G	1,712	1,653	50.0	OE	729,297	619,894
HNR-2	W G	823	798	49.0	O1-4	1,134,514	744,227
DOGS	W G	1,055	895	47.0	O2.5	2,443,179	570,159
SHIP-Trend	NE G	986	937	44.0	O2.5	2,390,395	609,778
FoCus	N G	645	606	41.9	OEE	1,084,745	618,824
**Merged**			3,934 (cases), 8,455 (controls)	30.8 (ca), 48.3 (ctrl)			503,259

table S1. QC of data set DE1.

Description of genotyping chips: OE = Illumina OmniExpress, O2.5 = Illumina Omni2.5, OE1-4 = Illumina Omni1-Quad, OEE = Illumina OmniExpressExome.

Cohort	Origin	Samples pre QC	Samples post QC	Male [%]	Chip	Variants pre QC	Variants post QC
DE2 cases	Germany (G)	1,002	954	27.1	660	594,392	529,596
GSK	SE G	861	818	32.6	550	522,008	504,905
popgen	N G	664	624	39.6	550	554,996	503,671
KORA-S4F4	SE G	481	414	50.0	550	561,461	504,939
MARS	SE G	87	84	65.5	610	716,385	518,203
**Merged**			954 (cases), 1,940 (controls)	27.1 (ca), 40.0 (ctrl)			456,558

table S2. QC of data set DE2.

Description of genotyping chips: 660 = Illumina Human660-Quad, 550 = Illumina 550k, 610 = Illumina Human610-Quad.

Population substructure

MDS components of the genetic similarity matrix were calculated in PLINK 1.90b3s according to the following steps:

- Filtering of genotyped variants:
  - Only autosomal chromosomes
  - MAF ≥ 0.05
  - HWE *p*-value ≥ 10^{-3}
- Removal of the extended MHC region (chr. 6, 25-35 Mbp) and a typical inversion site on chr. 8 (7-13 Mbp).
- Pruning by --*indep-pairwise 200 100 0.2*
- IBS/IBD computation using --*genome*
- Calculation of MDS components using the eigendecomposition-based algorithm

MDS components of the two data sets showed little evidence for heterogeneity (figs. S1 and S2).

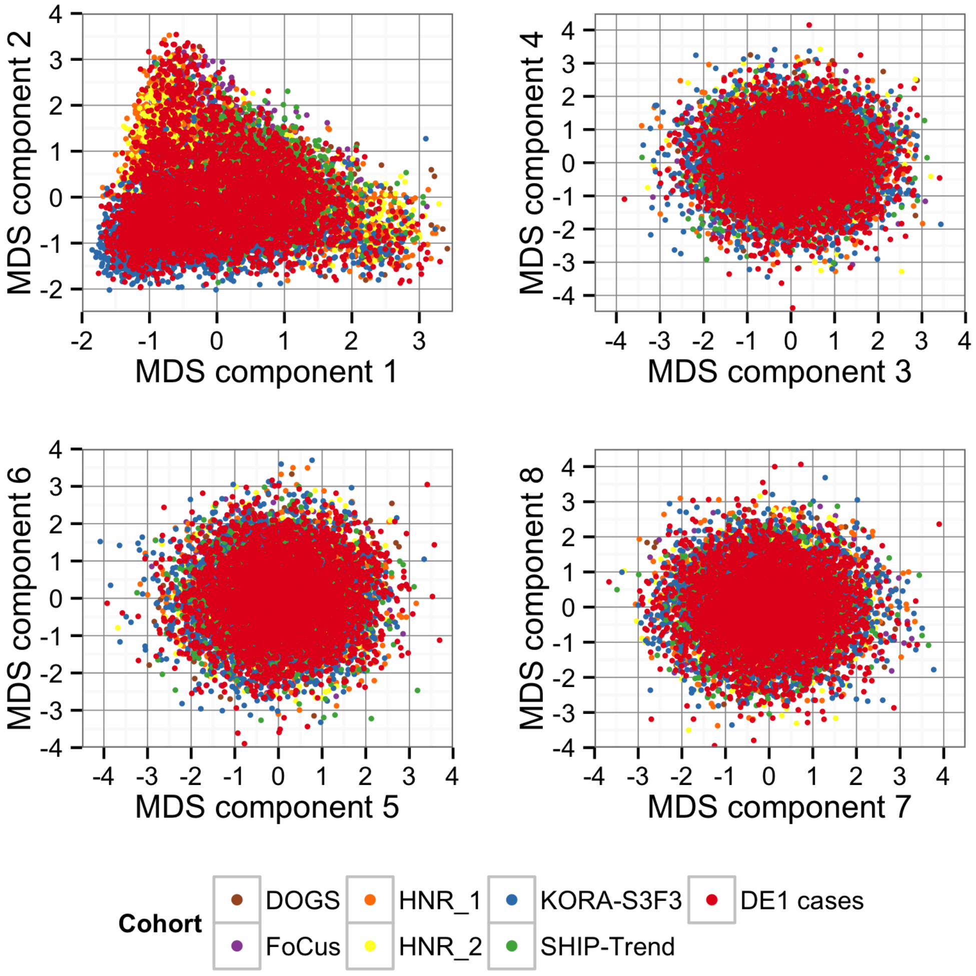

fig. S1. Substructure analysis results in DE1.
Scaled MDS components of the genetic similarity matrix of data set DE1.

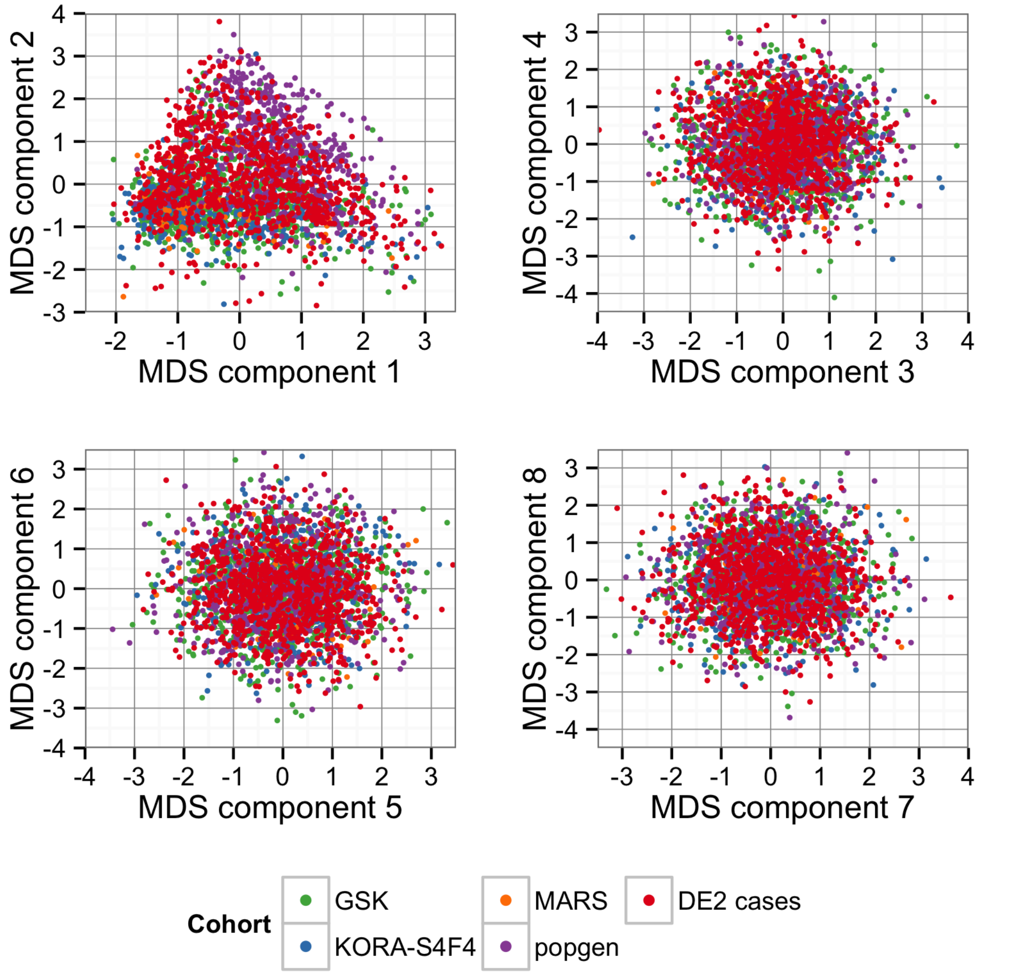

fig. S2. Substructure analysis results in DE2.
Scaled MDS components of the genetic similarity matrix of data set DE2.

Imputation of genotype data

Prior to imputation, genotypes were aligned to the 1000 genomes phase I reference panel (June 2014 release) using SHAPEIT v2 (r837) (*18*) and PLINK v1.90b3s. Subsequently, pre-phasing (haplotype estimation) was conducted for each chromosome separately using SHAPEIT (*17*).

Imputation was performed using IMPUTE2 v2.3.2 (*16*) in 5 Mbp chunks with 500 kbp buffers, filtering out variants that are monomorphic in the EUR samples. Chunks with < 51 genotyped variants or concordance rates < 92 % were fused with neighboring chunks and re-imputed. Imputed variants were filtered for MAF ≥ 1 %, INFO metric ≥ 0.8 and HWE *p*-value ≥ 10^{-6} using QCTOOL. After imputation and QC, data set DE1 contained 8,221,608 variants, DE2 8,143,088.

Imputation and analysis of *HLA* alleles

HLA alleles were imputed from genotyping data separately for DE1 and DE2 using HIBAG v1.6.0 (*20*). Alleles with a posterior probability >0.5 were converted to hard calls. After QC, imputed alleles were obtained for 3,966 cases and 8,329 controls from DE1 and DE2.

Imputation results were validated using *HLA* typing of 442 patients from DE1:

Allele	Accuracy (%)	Call rate (%)
HLA-A	96.8	99.0
HLA-B	95.5	94.7
HLA-C	96.7	98.7
DPB1	91.0	96.1
DQB1	96.6	98.8
DRB1	95.5	93.3
Median	96.1	97.4

Alleles with a frequency < 1% were excluded from further analyses. MDS components were recalculated for DE1 and DE2 based on the subset of individuals. Eight MDS components and sex were used as covariates for step-wise logistic regression in R. DE1 and DE2 were analyzed separately and results combined in a pooled analysis using fixed effects.

Age at onset

Age at onset was available for a subset of 1,519 patients from DE1. As the age at onset was not normally distributed, the phenotype was transformed using rank-based inverse normal transformation. MDS components were recalculated based on the subset of the data (fig. S3A). Both the age at onset and imputed *HLA* alleles were available for 1,196 cases.

Median-based genomic inflation of the GWAS (fig. S3B) was 1.007.

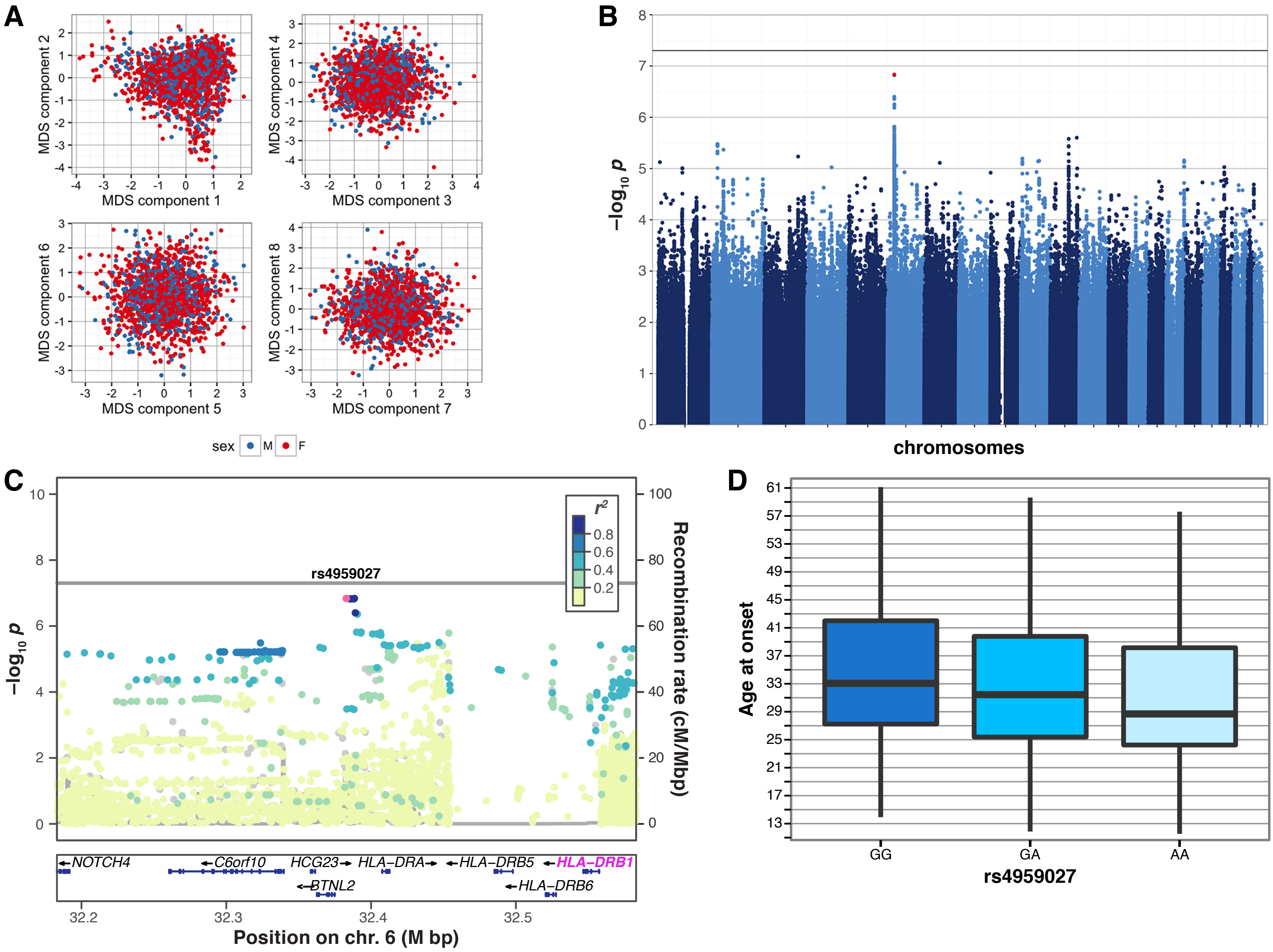

fig. S3. GWAS with age at onset.

A: Scaled MDS components of the subpopulation of 1,519 cases from DE1 of which the age at onset was available.

B: Manhattan plot showing strength of evidence for association with normalized age at onset. The grey line marks the genome-wide significance level and the red dot SNP rs4959027.

C: Locus-specific Manhattan plot for rs4959027. For a detailed description of this plot type see the legend of fig. S5.

D: Genotype of SNP rs4959027 *vs.* untransformed age at onset, two outliers were removed for better visibility.

Genome-wide association analysis

GWAS were performed on data sets DE1 and DE2 separately in PLINK 1.90b3s (*61*) using the dosage command (format 3) and sex as well as the first eight MDS components as covariates.

The median-based genomic inflation factor of the two data sets was as follows:

**DE1**	Overall	Without MHC	**DE2**	Overall	Without MHC
λ	1.100	1.088	λ	1.055	1.048
$\boldsymbol{\lambda_{1000,1000}}$	1.019	**1.016**	$\boldsymbol{\lambda_{1000,1000}}$	1.043	**1.037**
**Pooled**	Overall	Without MHC			
λ	1.126	1.114			
$\boldsymbol{\lambda_{1000,1000}}$	1.019	**1.017**			

table S3. Genomic inflation.

The extended MHC region was here defined as chr. 6, 27-35 Mbp. As λ scales with sample size, the genomic inflation rescaled to 1000 cases and 1000 controls ($\lambda_{1000,1000}$) is more informative (*19*).

Pooled analysis

Both data sets were combined in a fixed-effects pooled analysis using METASOFT v2.0.1 (*62*). 7,967,262 variants were present in both data sets.

15 loci outside the MHC region reached genome-wide significant *p*-values. The top variants showing the lowest *p*-value at each of these loci are listed in table S4.

All of these variants showed *p*-values $< 5\times10^{-6}$ in DE1 and $< 5\times10^{-8}$ in the pooled analysis. All showed lower *p*-values in the pooled analysis than in DE1.

Fig. S4 shows forest plots for all fifteen top variants ordered by position. Fig. S5 shows locus-specific Manhattan plots for all fifteen top variants ordered by position. Fig. S6 shows forest plots for the five variants replicated in Sardinians.

table S4. Genome-wide significant loci.

See supplementary file. All *p*-values shown in the table are two-sided. Gene names of known loci are as listed by Sawcer and colleagues (*3*).

Functional analysis

Examination of ENCODE regulation transcription factor ChIP-seq data (*70-72*) at the five loci examined in detail revealed that a strongly associated variant at the *DLEU1* locus (rs9591325) maps to a region to which many transcription factors bind (fig. S8).

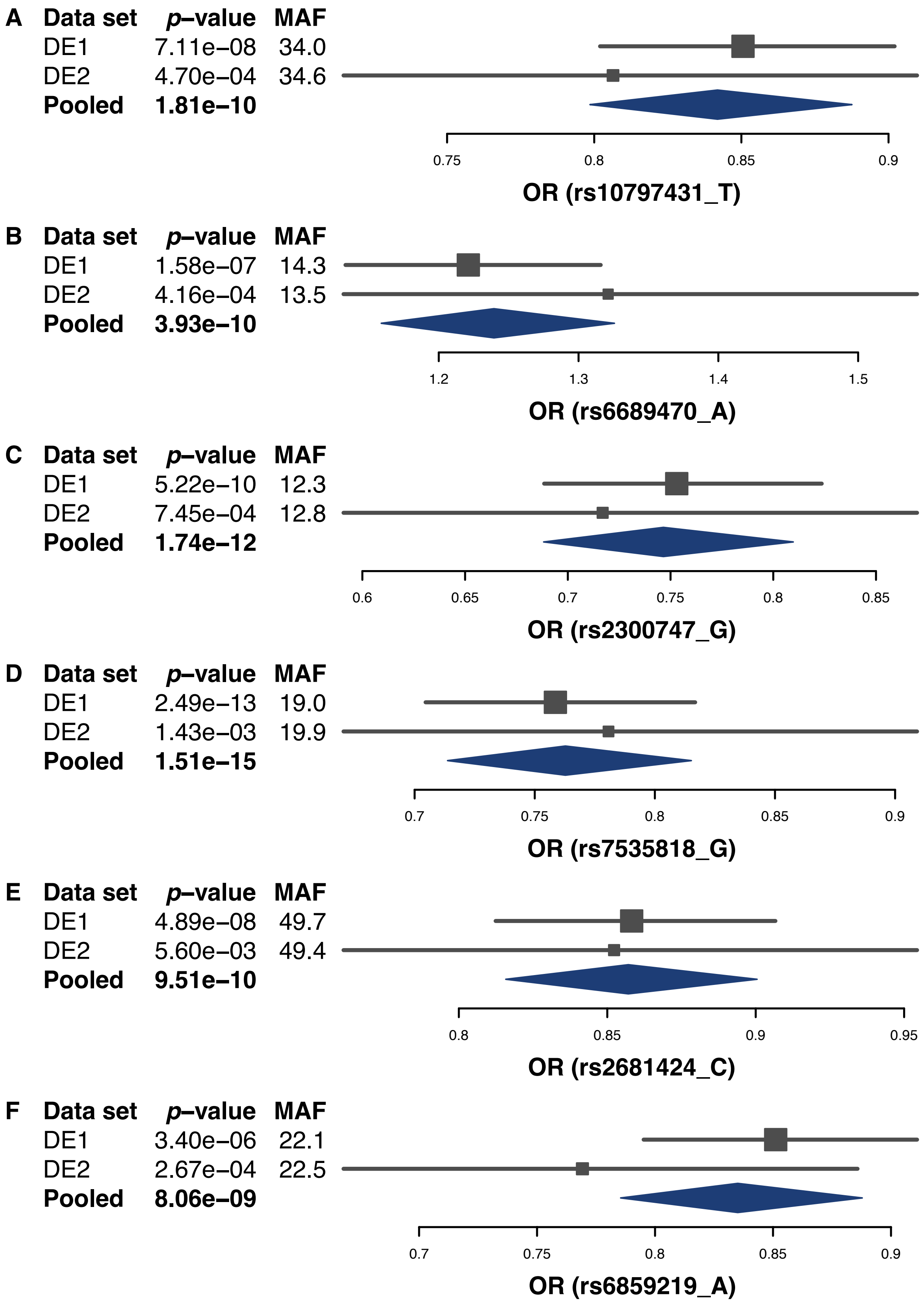
A Data set p–value MAF
DE1 7.11e–08 34.0
DE2 4.70e–04 34.6
Pooled 1.81e–10
0.75 0.8 0.85 0.9
OR (rs10797431_T)
B Data set p–value MAF
DE1 1.58e–07 14.3
DE2 4.16e–04 13.5
Pooled 3.93e–10
1.2 1.3 1.4 1.5
OR (rs6689470_A)
C Data set p–value MAF
DE1 5.22e–10 12.3
DE2 7.45e–04 12.8
Pooled 1.74e–12
0.6 0.65 0.7 0.75 0.8 0.85
OR (rs2300747_G)
D Data set p–value MAF
DE1 2.49e–13 19.0
DE2 1.43e–03 19.9
Pooled 1.51e–15
0.7 0.75 0.8 0.85 0.9
OR (rs7535818_G)
E Data set p–value MAF
DE1 4.89e–08 49.7
DE2 5.60e–03 49.4
Pooled 9.51e–10
0.8 0.85 0.9 0.95
OR (rs2681424_C)
F Data set p–value MAF
DE1 3.40e–06 22.1
DE2 2.67e–04 22.5
Pooled 8.06e–09
0.7 0.75 0.8 0.85 0.9
OR (rs6859219_A)

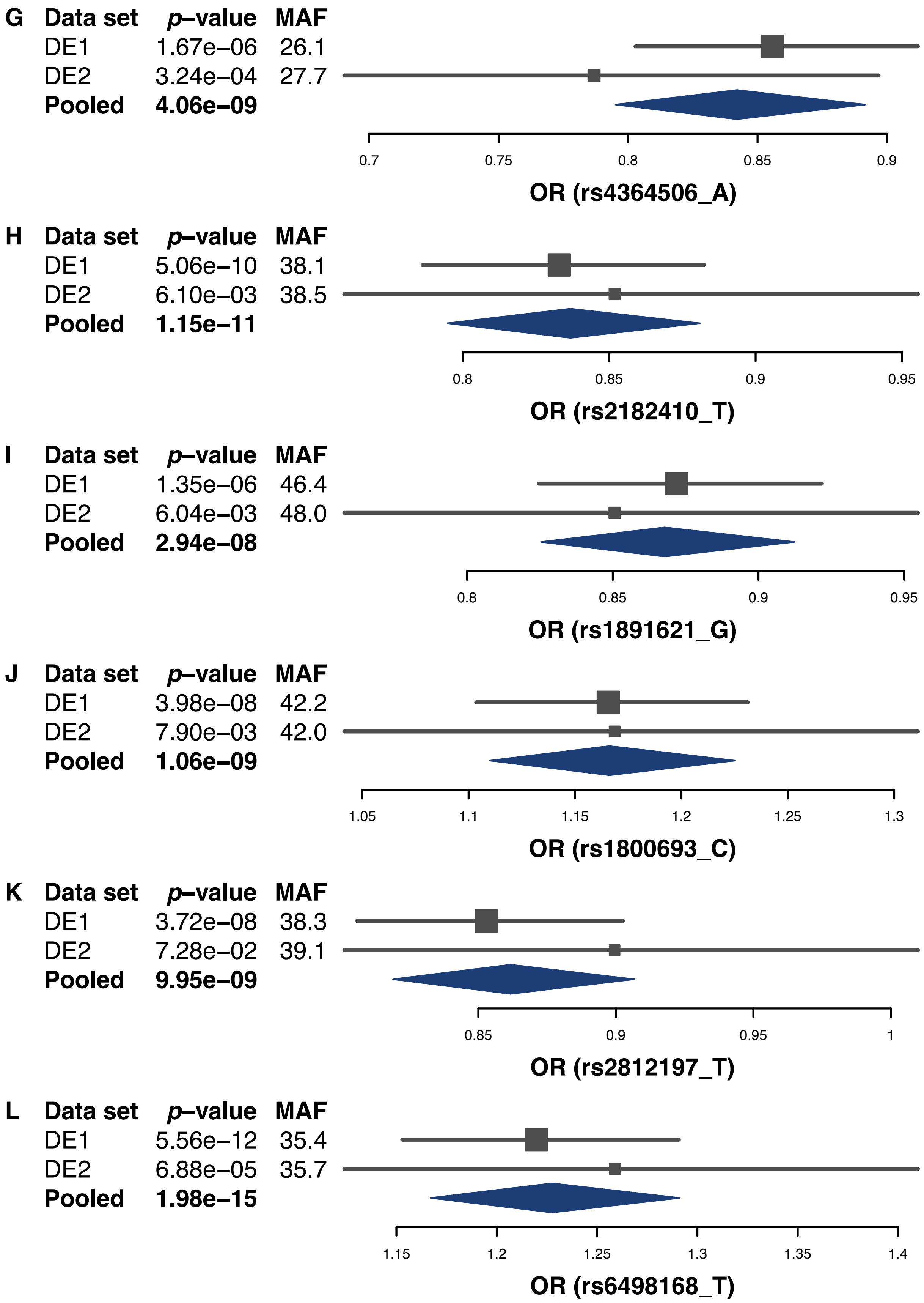
G
Data set
p–value
MAF
DE1
1.67e–06
26.1
DE2
3.24e–04
27.7
Pooled
4.06e–09
0.7
0.75
0.8
0.85
0.9
OR (rs4364506_A)
H
Data set
p–value
MAF
DE1
5.06e–10
38.1
DE2
6.10e–03
38.5
Pooled
1.15e–11
0.8
0.85
0.9
0.95
OR (rs2182410_T)
I
Data set
p–value
MAF
DE1
1.35e–06
46.4
DE2
6.04e–03
48.0
Pooled
2.94e–08
0.8
0.85
0.9
0.95
OR (rs1891621_G)
J
Data set
p–value
MAF
DE1
3.98e–08
42.2
DE2
7.90e–03
42.0
Pooled
1.06e–09
1.05
1.1
1.15
1.2
1.25
1.3
OR (rs1800693_C)
K
Data set
p–value
MAF
DE1
3.72e–08
38.3
DE2
7.28e–02
39.1
Pooled
9.95e–09
0.85
0.9
0.95
1
OR (rs2812197_T)
L
Data set
p–value
MAF
DE1
5.56e–12
35.4
DE2
6.88e–05
35.7
Pooled
1.98e–15
1.15
1.2
1.25
1.3
1.35
1.4
OR (rs6498168_T)

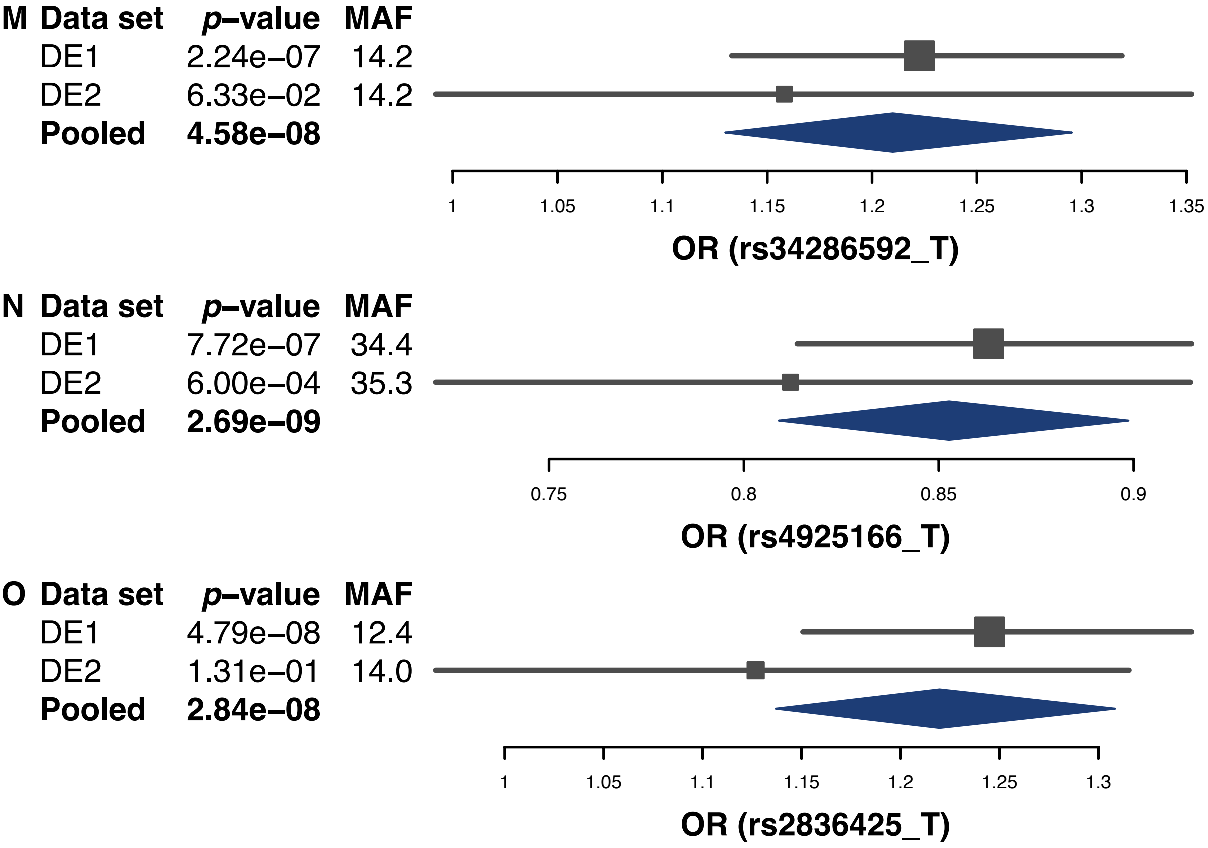

fig. S4. Forest plots of all non-MHC top genome-wide significant variants. Variants are ordered by chromosome and position. All *p*-values are two-sided, the MAF is based on controls (in %). ORs are relative to the minor allele.

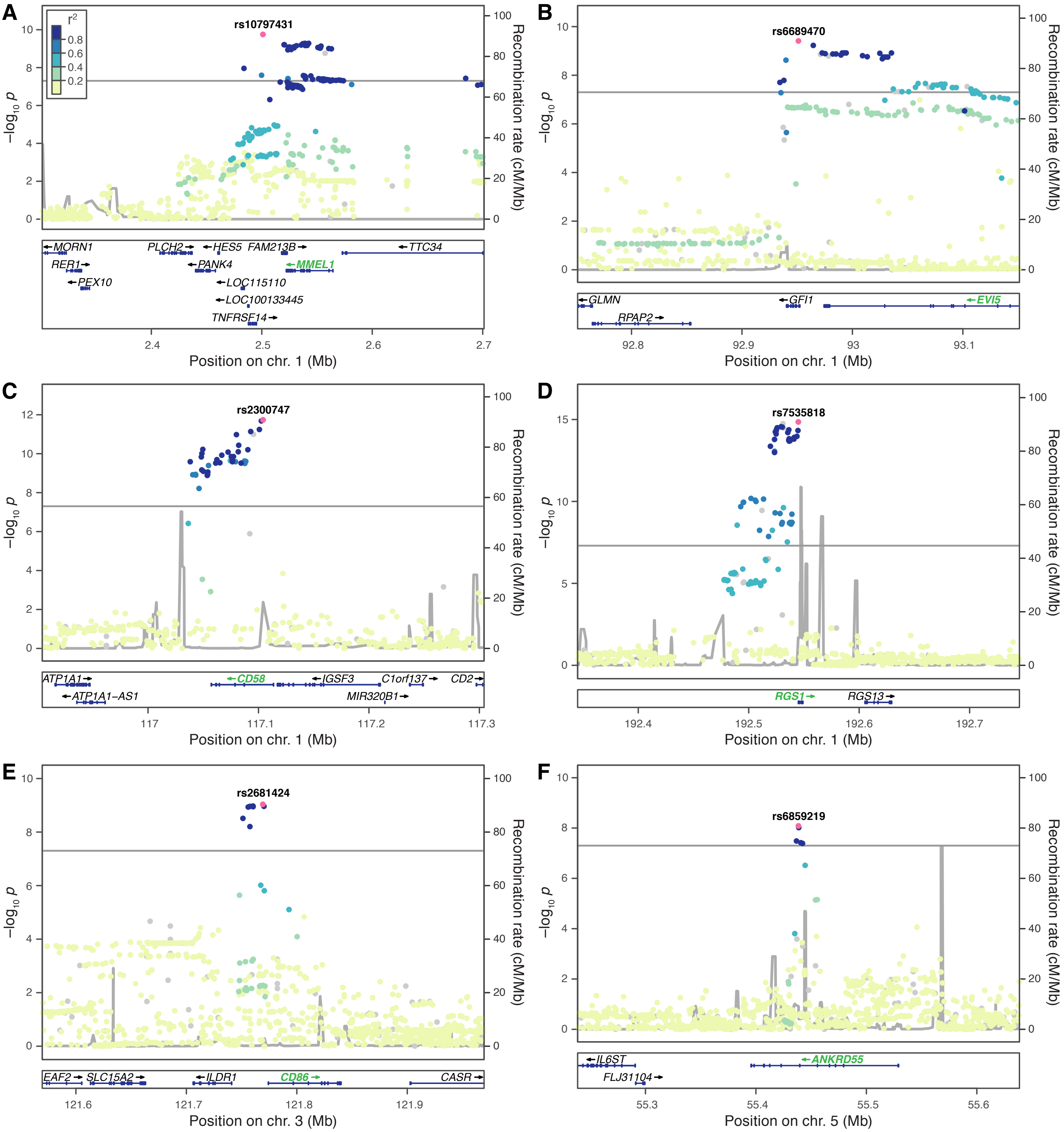
A
r2
0.8
0.6
0.4
0.2
rs10797431
MORN1
RER1
PEX10
PLCH2
PANK4
HES5
FAM213B
LOC115110
LOC100133445
TNFRSF14
MMEL1
TTC34
Position on chr. 1 (Mb)
B
rs6689470
GLMN
RPAP2
GFI1
EVI5
Position on chr. 1 (Mb)
C
rs2300747
ATP1A1
ATP1A1−AS1
CD58
IGSF3
C1orf137
MIR320B1
CD2
Position on chr. 1 (Mb)
D
rs7535818
RGS1
RGS13
Position on chr. 1 (Mb)
E
rs2681424
EAF2
SLC15A2
ILDR1
CD86
CASR
Position on chr. 3 (Mb)
F
rs6859219
IL6ST
FLJ31104
ANKRD55
Position on chr. 5 (Mb)
−log10 p
Recombination rate (cM/Mb)

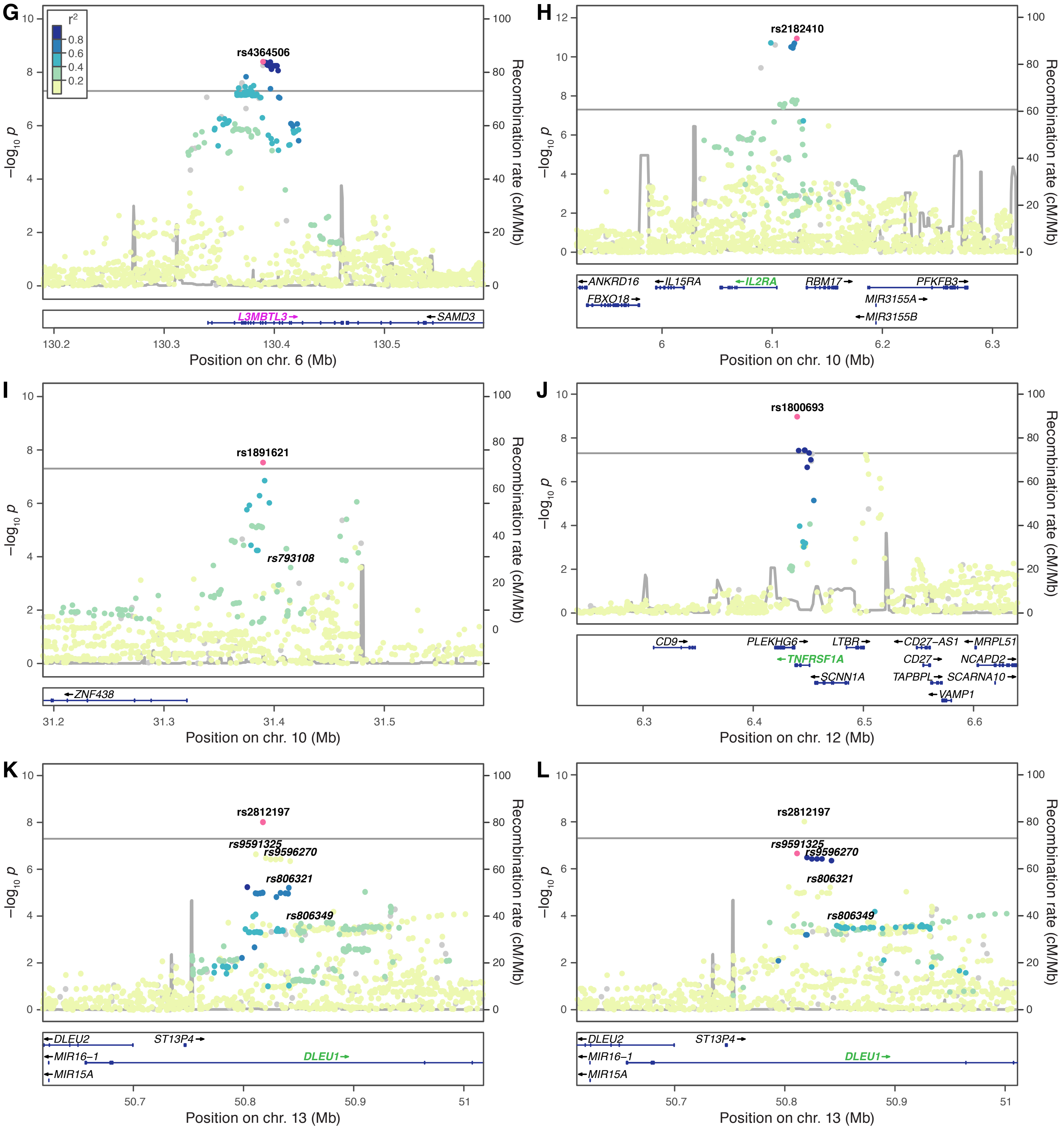
G
H
I
J
K
L
r2
0.8
0.6
0.4
0.2
rs4364506
L3MBTL3
SAMD3
Position on chr. 6 (Mb)
rs2182410
ANKRD16
IL15RA
IL2RA
RBM17
PFKFB3
FBXO18
MIR3155A
MIR3155B
Position on chr. 10 (Mb)
rs1891621
rs793108
ZNF438
Position on chr. 10 (Mb)
rs1800693
CD9
PLEKHG6
LTBR
CD27−AS1
MRPL51
TNFRSF1A
CD27
NCAPD2
SCNN1A
TAPBPL
SCARNA10
VAMP1
Position on chr. 12 (Mb)
rs2812197
rs9591325
rs9596270
rs806321
rs806349
DLEU2
ST13P4
MIR16−1
DLEU1
MIR15A
Position on chr. 13 (Mb)
−log10 p
Recombination rate (cM/Mb)

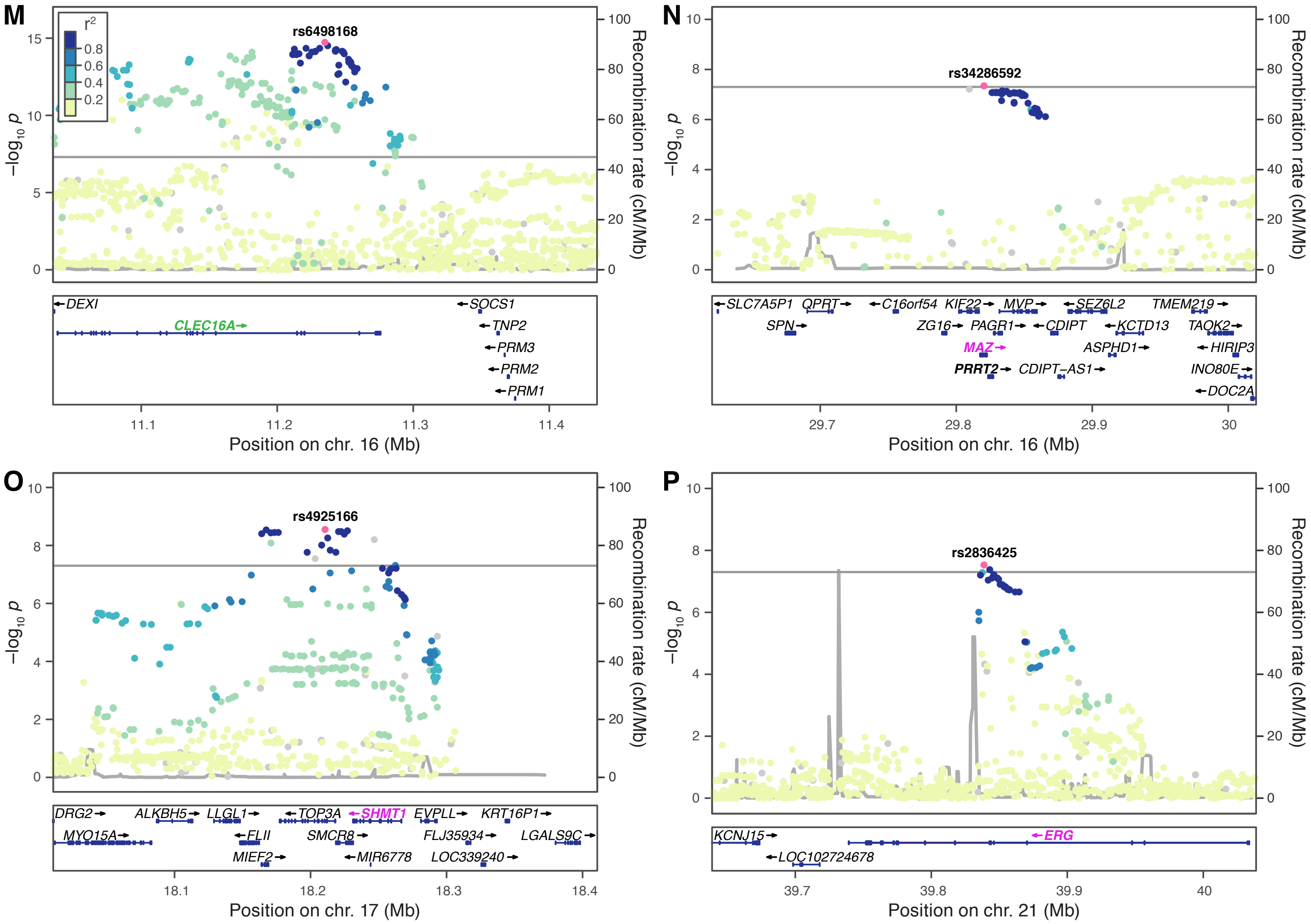

fig. S5. Locus-specific Manhattan plots.

Plots of genome-wide significant loci outside the MHC region were generated using LocusZoom with EUR samples of the 1000 genomes March 2012 reference panel on the hg19 build. Color of dots indicates LD with the lead variant (shown in pink). Grey dots represent signals with missing r^2 values. The most plausible candidate gene in each associated region is indicated in green for known loci and in magenta for novel loci. For the intergenic locus (I) the previously published SNP is indicated in italics. Two separate plots were generated for the *DLEU1* locus (K,L), using the two variants with lowest *p*-values, as they were in poor LD with each other ($r^2 = 0.14$).

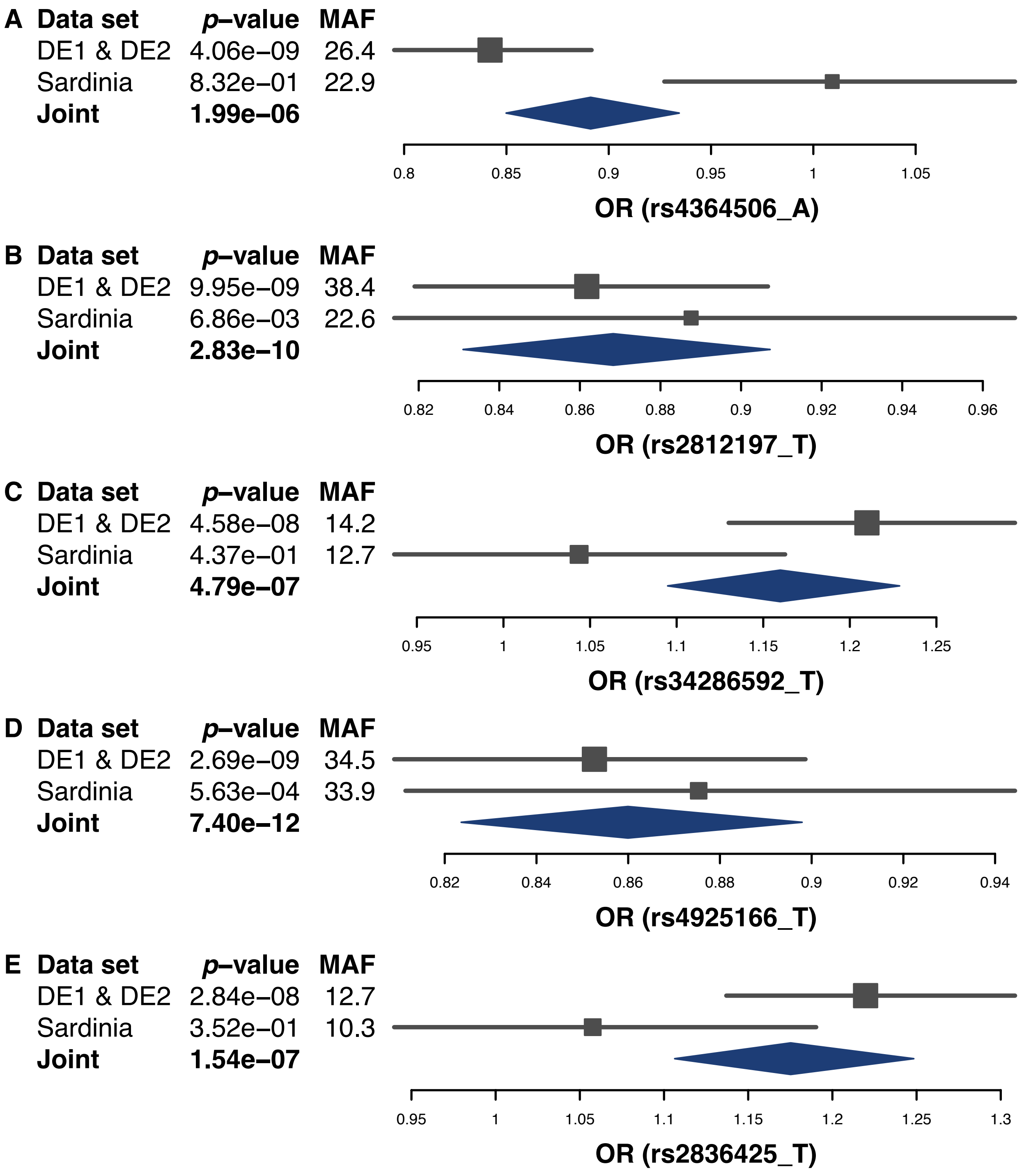

fig. S6. Forest plots of novel variants replicated in a Sardinian cohort. Variants are ordered by chromosome and position. All *p*-values are two-sided, the MAF is based on controls (in %). ORs are relative to the minor allele.

Expression and methylation QTL analysis

Data sets

DE1

This cohort consists of a subset of 242 patients from data set DE1 (73 male, 169 female). This cohort was described in the sample section at the beginning of this document. The majority of patients was treatment-naïve.

MPIP

The group of subjects consists of 289 Caucasian individuals (196 male, 93 female) recruited by the Max Planck Institute of Psychiatry (MPIP). Recruitment strategies and further characterization of this cohort have been described previously (*25*). 160 of them were healthy (115 men, 45 women), 129 were treated for depressive disorder (81 men, 48 women). The local ethics committee has approved the study and all individuals gave written informed consent.

GTP

These 328 African American subjects (99 male, 229 female) that all have experienced stressful life events constitute a subset of the GTP (Grady Trauma Project) cohort. Details on the cohort have been described previously (*26*-*28*). All participants provided written informed consent and all procedures were approved by the Institutional Review Boards of the Emory University School of Medicine and Grady Memorial Hospital.

Methods

DE1: Gene expression

Whole blood RNA was collected using Tempus Blood RNA Tubes (Applied Biosystems, Foster City, CA), followed by quality control (RIN) and quantification using the Agilent Bioanalyzer. RNA was amplified using the Illumina TotalPrep-96 RNA Amplification kit (Illumina, San Diego, CA). The RNA was hybridized to Illumina HT-12 v4 expression BeadChips. Raw probe intensities were exported using Illumina's GenomeStudio and further statistical processing was carried out using R version 3.2.1. Quality control on background-corrected bead level data was performed using the Bioconductor/R package beadarray (*64*). Arrays that showed both a low signal-to-noise ratio (< 6 fold) and < 80 % of housekeeping transcripts significantly expressed above background level were removed from further analysis. After removal of outliers (probes outside the range interval median ± 3 median absolute deviations), probe intensities were summarized by calculating average intensities and standard deviations for each probe and feature. Detection p-values were calculated on the bead summary data. Further pre-processing was conducted using the Bioconductor/R packages lumi (*65*) and vsn (*66*). Each probe was transformed and normalized through variance stabilization and normalization (VSN). Probes which showed a detection p-value < 0.05 in more than 10% of the samples, which could not be mapped to a known transcript, or which were identified as cross-

hybridizing the Re-Annotator pipeline (*67*) were removed. This left 20,302 transcripts from 242 samples. Technical batch effects were identified by inspecting the association of the first two principal components of expression levels with amplification round, amplification plate, amplification plate column and row, as well as with expression chip. The data were then adjusted using ComBat (*68*).

MPIP: Gene expression, DNA methylation and genotyping

Processing of gene expression, DNA methylation, and genotyping data for this cohort has been described previously (*25*, *28*). In summary, for gene expression, baseline whole blood RNA of the MPIP cohort was collected using PAXgene Blood RNA Tubes (PreAnalytiX, Hombrechtikon, Switzerland), the RNA hybridized to Illumina HT-12 v3 and v4 expression BeadChips (Illumina, San Diego, CA), followed by quality control conducted in R version 3.2.1. For DNA methylation data, genomic DNA was extracted from whole blood using the Gentra Puregene Blood Kit (Qiagen, Valencia, CA) and bisulfite-converted using the Zymo EZ-96 DNA Methylation Kit (Zymo Research, Irvine, CA). DNA methylation levels were assessed using the Illumina HumanMethylation450 BeadChip array. Genotyping was performed using Illumina Human610-Quad (n=173) and OmniExpress (n=120) genotyping BeadChips. Genotypes were imputed as described for DE1 and DE2. Combined gene expression, DNA methylation, and genotyping data were available for a subset of 223 subjects.

GTP: Gene expression, DNA methylation and genotyping

Processing of gene expression (*27*), DNA methylation (*27*, *28*), and genotyping data (*26*) has been described previously and was conducted in an analogous manner to the MPIP cohort. DNA was extracted from whole blood, samples were genotyped using Illumina Omni1-Quad and OmniExpress BeadChip arrays, gene expression was determined using Illumina HT-12 v3.0 BeadChips and DNA methylation using the Illumina HumanMethylation450 BeadChip.

For DNA methylation data, intensity read outs, normalization, cell type composition estimation, beta and M-value calculation were conducted using the R minfi package v1.10.2 (*73*). Probes were excluded if detection *p*-value > 0.01 in at least 75 % of samples. Probes located within 10 bp of a SNP with MAF ≥ 0.05 as well as non-specific binding probes were excluded as well. Data were normalized using functional normalization (*74*). Batch effects (array and position) were removed using ComBat (*68*). Combined gene expression, DNA methylation, and genotyping data were available for a subset of 279 individuals.

eQTL analysis

For each of the 15 genome-wide significant loci, all 429 transcripts beginning or ending within 1 Mbp up- or downstream of a lead variant were determined. Associations between genotype and expression levels were determined in data set DE1 by linear regression, using sex, age, and 3 MDS components as covariates. To correct for multiple testing, *p*-values were first corrected for the number of transcripts per *cis* window, followed by calculation of the false discovery rate (FDR) for the total number of variants tested.

Replication of eQTLs with an FDR < 0.05 in data set DE1 was conducted in control cohorts MPIP and GTP. For MPIP, the covariates sex, age, BMI, disease status, and 3 MDS components were used in linear regression. For GTP, covariates were sex, age, and 4 MDS components. eQTLs were also looked up in the GTEx database (*29*). Here, only associations in whole blood were considered.

The four eQTLs with FDRs < 0.05 are listed in table S5. One eQTL (rs4925166 and *SHMT1*) was significant in all data sets (fig. S7). The three other eQTLs were each significant in at least one of the control cohorts (rs10797431 and *MMEL1*, rs6859219 and *ANKRD55*, rs6859219 and *ANKRD55).*

table S5. eQTLs with FDR < 0.05 in data set DE1.

See supplementary file. All *p*-values shown in the table are two-sided.

DNA methylation analysis

210 CpG probes were identified in data set MPIP that mapped to *SHMT1*. After removing the quartile of probes showing the lowest variation in methylation status, 157 CpGs remained. Association of DNA methylation with imputed genotype was assessed by linear regression, using sex, age, BMI, disease status, 3 MDS components, and estimated cell counts (*75*) as covariates.

The eight CpG probes showing an FDR < 0.05 in the MPIP data set were examined in data set GTP, using sex, age, 4 MDS components, and estimated cell counts as covariates. Replicated CpG sites are shown in table S6.

CpG	MPIP			GTP		
	**Effect**	***p*-value**	**FDR**	**Effect**	***p*-value**	**FDR**
cg26763362	-0.03	3.21e-20	5.04e-18	-0.03	1.98e-14	1.58e-13
cg02426414	-0.01	3.04e-07	2.39e-05	-0.01	3.03e-06	1.21e-05
cg02116225	-0.01	2.98e-04	1.17e-02	-0.01	1.31e-05	3.49e-05

table S6. Replicated mQTLs of rs4925166 and CpG sites in *SHMT1*.

Mediation analysis

In order to test the hypothesis rs4925166 → cg26763362 → ILMN_1811933 (probe for *SHMT1* transcript), mediation analysis was conducted (table S7).

A: Data set MPIP:

Step	Model	Effect	p-value	Outcome
1	ILMN_181193 ~ rs4925166	0.20	8.33e-10	Association
2	cg26763362 ~ rs4925166	-0.03	1.39e-20	Association
3	ILMN_181193 ~ cg26763362	-3.88	1.76e-10	Association
4	ILMN_181193 ~ rs4925166 +cg26763362	0.13	2.78e-03	Association
5	Indirect effect: Step 1 – Step 4	0.07		

B: Data set GTP:

Step	Model	Effect	p-value	Outcome
1	ILMN_181193 ~ rs4925166	0.11	3.12e-04	Association
2	cg26763362 ~ rs4925166	-0.03	2.05e-12	Association
3	ILMN_181193 ~ cg26763362	-1.43	7.56e-05	Association
4	ILMN_181193 ~ rs4925166 +cg26763362	0.07	1.79e-02	Association
5	Indirect effect: Step 1 – Step 4	0.04		

table S7. Mediation analysis.

This analysis demonstrates that partial mediation takes place.

All steps were conducted with additional covariates as described above. Note that the observed effects differ from the ones described for the expression analysis, as a lower number of samples contained both expression and methylation data.

The R package mediation (*30*) allows for causal mediation analysis including nonparametric bootstrap for estimation of confidence intervals and *p*-values. The analysis confirms that partial mediation takes place (Fig. 3, table S8).

Type	MPIP		GTP	
	**Effect**	***p*-value**	**Effect**	***p*-value**
Total effect	0.197	0	0.111	1.5e-04
Direct effect	0.127	1.9e-04	0.074	1.7e-02
Causal mediation effect	0.070	3.7e-04	0.037	6.6e-03
Proportion mediated	0.355	3.7e-04	0.332	6.6e-03

table S8. Causal mediation analysis.

Results were obtained using 1,000,000 simulations. The *p*-value for the total effect in MPIP is 0, as this number of simulations is not sufficient to estimate a *p*-value as low as 8.33×10^{-10} (table S7A). Note that the effects and *p*-values observed here differ from the ones shown in Table 4, as a lower number of samples contained both expression and methylation data than expression data alone.

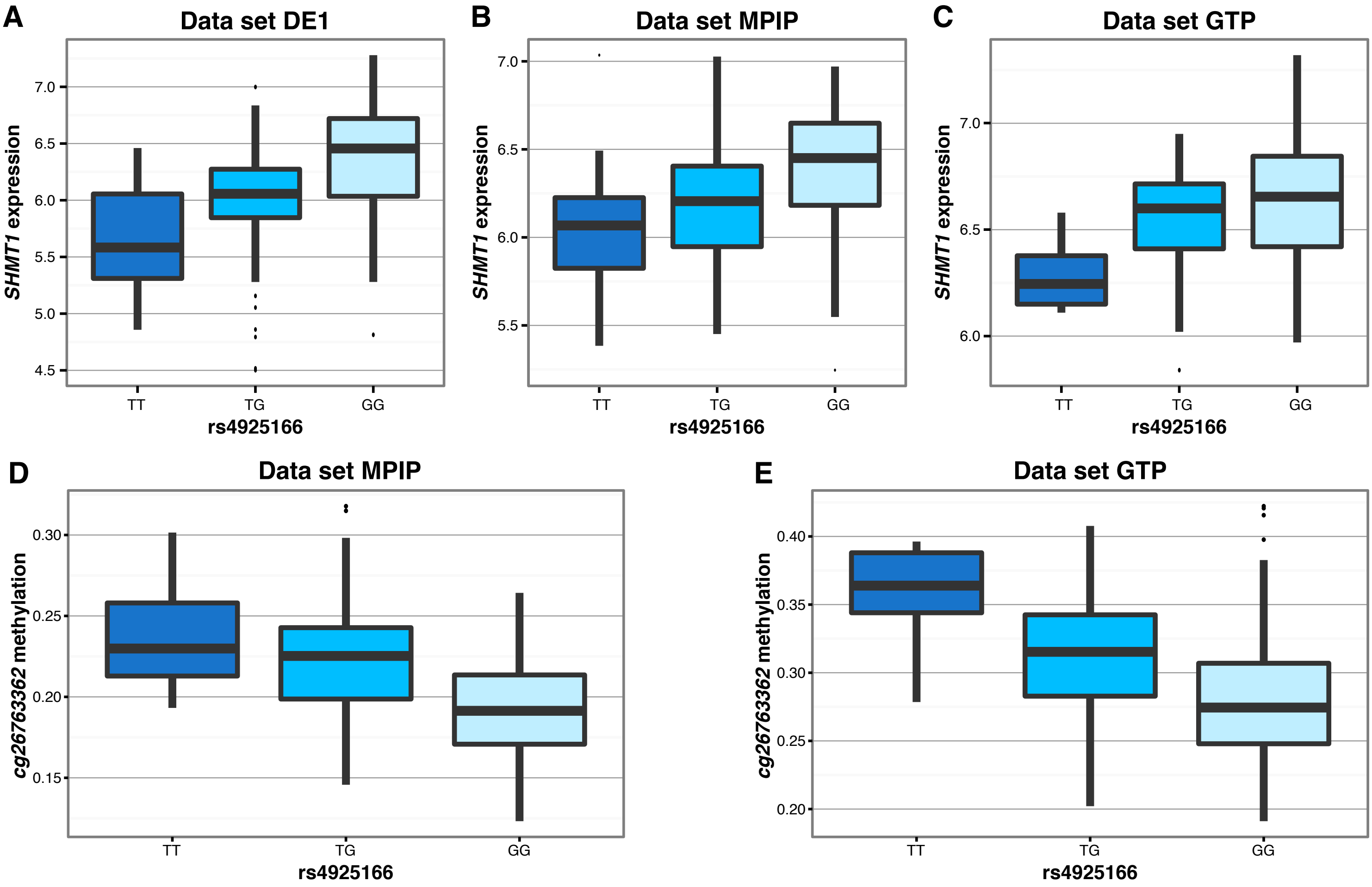

fig. S7. eQTL and mQTL analysis for rs4925166.
A: eQTL between rs4925166 and *SHMT1* (ILMN_1811933). **B:** Replication of this eQTL in control data set MPIP. **C:** Replication of this eQTL in control data set GTP.
D: mQTL between rs4925166 and cg26763362 within *SHMT1* in MPIP controls.
E: Replication of this mQTL in control data set GTP.

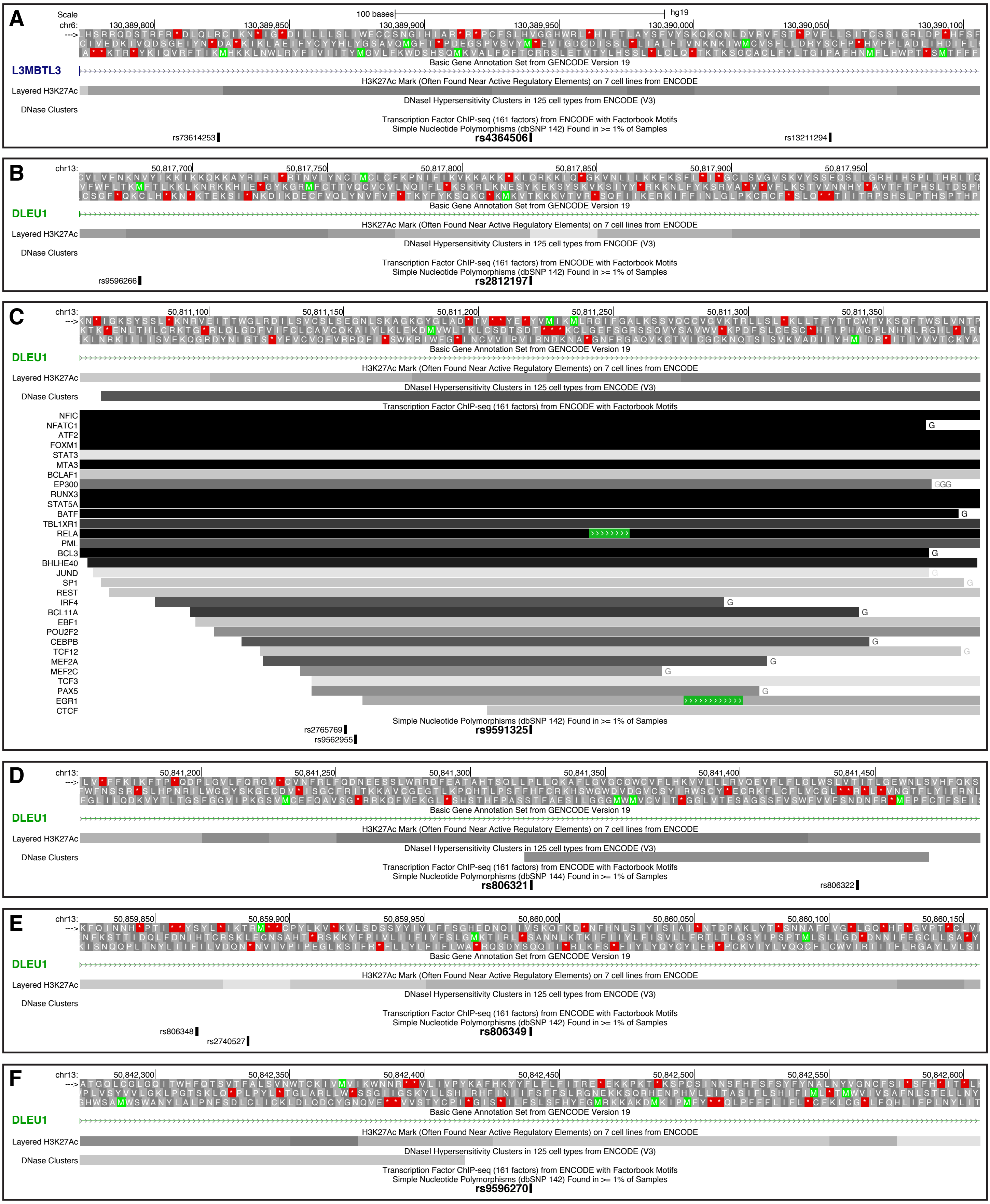
A
Scale
chr6:
100 bases
hg19
130,389,800
130,389,850
130,389,900
130,389,950
130,390,000
130,390,050
130,390,100
L3MBTL3
Basic Gene Annotation Set from GENCODE Version 19
Layered H3K27Ac
H3K27Ac Mark (Often Found Near Active Regulatory Elements) on 7 cell lines from ENCODE
DNase Clusters
DNaseI Hypersensitivity Clusters in 125 cell types from ENCODE (V3)
Transcription Factor ChIP-seq (161 factors) from ENCODE with Factorbook Motifs
Simple Nucleotide Polymorphisms (dbSNP 142) Found in >= 1% of Samples
rs73614253
rs4364506
rs13211294
B
chr13:
50,817,700
50,817,750
50,817,800
50,817,850
50,817,900
50,817,950
DLEU1
Basic Gene Annotation Set from GENCODE Version 19
Layered H3K27Ac
H3K27Ac Mark (Often Found Near Active Regulatory Elements) on 7 cell lines from ENCODE
DNase Clusters
DNaseI Hypersensitivity Clusters in 125 cell types from ENCODE (V3)
Transcription Factor ChIP-seq (161 factors) from ENCODE with Factorbook Motifs
Simple Nucleotide Polymorphisms (dbSNP 142) Found in >= 1% of Samples
rs9596266
rs2812197
C
chr13:
50,811,100
50,811,150
50,811,200
50,811,250
50,811,300
50,811,350
DLEU1
Basic Gene Annotation Set from GENCODE Version 19
Layered H3K27Ac
H3K27Ac Mark (Often Found Near Active Regulatory Elements) on 7 cell lines from ENCODE
DNase Clusters
DNaseI Hypersensitivity Clusters in 125 cell types from ENCODE (V3)
Transcription Factor ChIP-seq (161 factors) from ENCODE with Factorbook Motifs
NFIC
NFATC1
ATF2
FOXM1
STAT3
MTA3
BCLAF1
EP300
RUNX3
STAT5A
BATF
TBL1XR1
RELA
PML
BCL3
BHLHE40
JUND
SP1
REST
IRF4
BCL11A
EBF1
POU2F2
CEBPB
TCF12
MEF2A
MEF2C
TCF3
PAX5
EGR1
CTCF
Simple Nucleotide Polymorphisms (dbSNP 142) Found in >= 1% of Samples
rs2765769
rs9562955
rs9591325
D
chr13:
50,841,200
50,841,250
50,841,300
50,841,350
50,841,400
50,841,450
DLEU1
Basic Gene Annotation Set from GENCODE Version 19
Layered H3K27Ac
H3K27Ac Mark (Often Found Near Active Regulatory Elements) on 7 cell lines from ENCODE
DNase Clusters
DNaseI Hypersensitivity Clusters in 125 cell types from ENCODE (V3)
Transcription Factor ChIP-seq (161 factors) from ENCODE with Factorbook Motifs
Simple Nucleotide Polymorphisms (dbSNP 144) Found in >= 1% of Samples
rs806321
rs806322
E
chr13:
50,859,850
50,859,900
50,859,950
50,860,000
50,860,050
50,860,100
50,860,150
DLEU1
Basic Gene Annotation Set from GENCODE Version 19
Layered H3K27Ac
H3K27Ac Mark (Often Found Near Active Regulatory Elements) on 7 cell lines from ENCODE
DNase Clusters
DNaseI Hypersensitivity Clusters in 125 cell types from ENCODE (V3)
Transcription Factor ChIP-seq (161 factors) from ENCODE with Factorbook Motifs
Simple Nucleotide Polymorphisms (dbSNP 142) Found in >= 1% of Samples
rs806348
rs2740527
rs806349
F
chr13:
50,842,300
50,842,350
50,842,400
50,842,450
50,842,500
50,842,550
50,842,600
DLEU1
Basic Gene Annotation Set from GENCODE Version 19
Layered H3K27Ac
H3K27Ac Mark (Often Found Near Active Regulatory Elements) on 7 cell lines from ENCODE
DNase Clusters
DNaseI Hypersensitivity Clusters in 125 cell types from ENCODE (V3)
Transcription Factor ChIP-seq (161 factors) from ENCODE with Factorbook Motifs
Simple Nucleotide Polymorphisms (dbSNP 142) Found in >= 1% of Samples
rs9596270

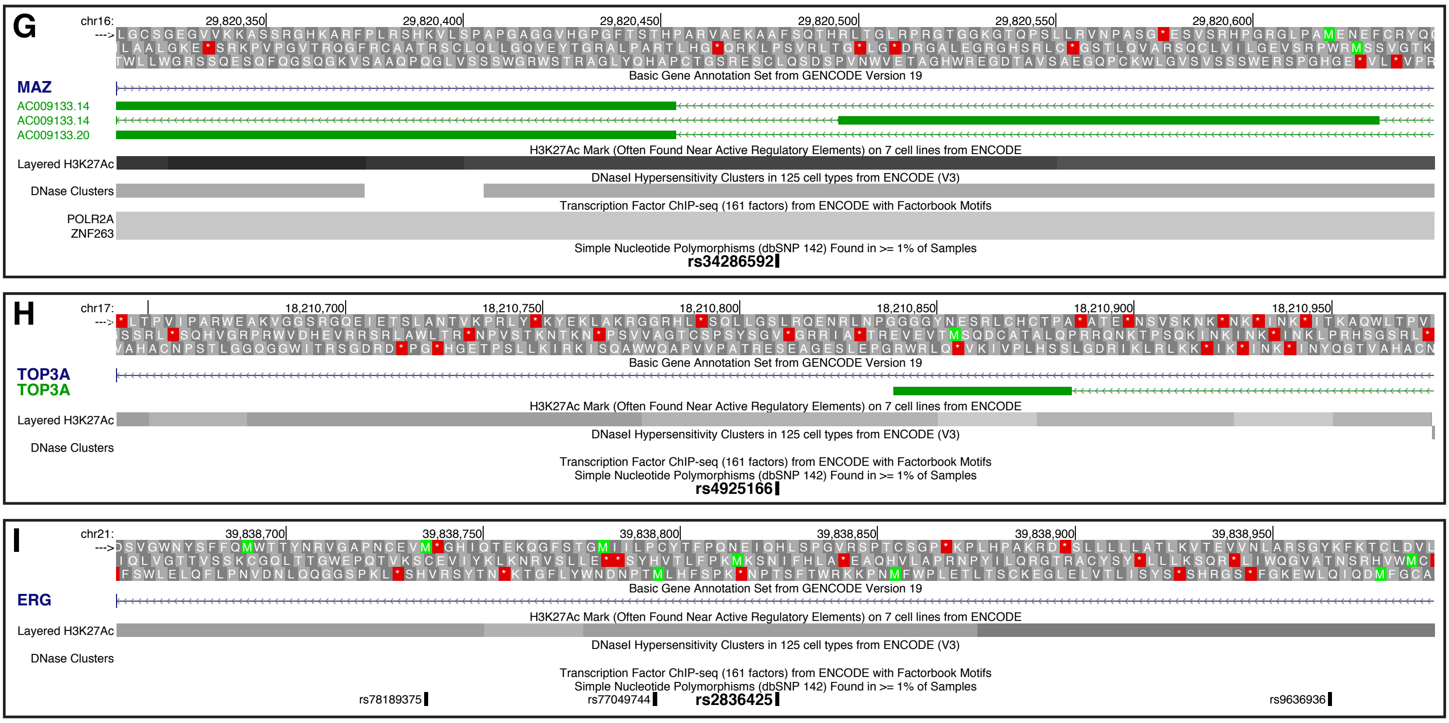

fig. S8. Transcription factor binding sites.
Based on Encode regulation transcription factor ChIP-seq data.
A: rs4364506 (*L3MBTL3*); **B:** rs2812197 (*DLEU1*), **C:** rs9591325 (*DLEU1*), **D:** rs806321 (*DLEU1*), **E:** rs806349 (*DLEU1*), **F:** rs9596270 (*DLEU1*); **G:** rs34286592 (*MAZ*); **H:** rs4925166 (*SHMT1*); **I:** rs2836425 (*ERG*).
